# Functional roles for PIEZO1 and PIEZO2 in urothelial mechanotransduction and lower urinary tract interoception

**DOI:** 10.1172/jci.insight.152984

**Published:** 2021-10-08

**Authors:** Marianela G. Dalghi, Wily G. Ruiz, Dennis R. Clayton, Nicolas Montalbetti, Stephanie L. Daugherty, Jonathan M. Beckel, Marcelo D. Carattino, Gerard Apodaca

**Affiliations:** 1 Department of Medicine,; 2 Department of Pharmacology and Chemical Biology, and; 3 Department of Cell Biology, University of Pittsburgh School of Medicine, Pittsburgh, Pennsylvania, USA.

**Keywords:** Cell Biology, Epithelial transport of ions and water, Signal transduction, Urology

## Abstract

The mechanisms that link visceral mechanosensation to the perception of internal organ status (i.e., interoception) remain elusive. In response to bladder filling, the urothelium releases ATP, which is hypothesized to stimulate voiding function by communicating the degree of bladder fullness to subjacent tissues, including afferent nerve fibers. To determine if PIEZO channels function as mechanosensors in these events, we generated conditional urothelial *Piezo1*-, *Piezo2*-, and dual *Piezo1/2*-knockout (KO) mice. While functional PIEZO1 channels were expressed in all urothelial cell layers, *Piezo1*-KO mice had a limited phenotype. *Piezo2* expression was limited to a small subset of superficial umbrella cells, yet male *Piezo2*-KO mice exhibited incontinence (i.e., leakage) when their voiding behavior was monitored during their active dark phase. Dual *Piezo1/2*-KO mice had the most affected phenotype, characterized by decreased urothelial responses to mechanical stimulation, diminished ATP release, bladder hypoactivity in anesthetized *Piezo1/2*-KO females but not males, and urinary incontinence in both male and female *Piezo1/2*-KO mice during their dark phase but not inactive light one. Our studies reveal that the urothelium functions in a sex- and circadian rhythm–dependent manner to link urothelial PIEZO1/2 channel–driven mechanotransduction to normal voiding function and behavior, and in the absence of these signals, bladder dysfunction ensues.

## Graphical abstract

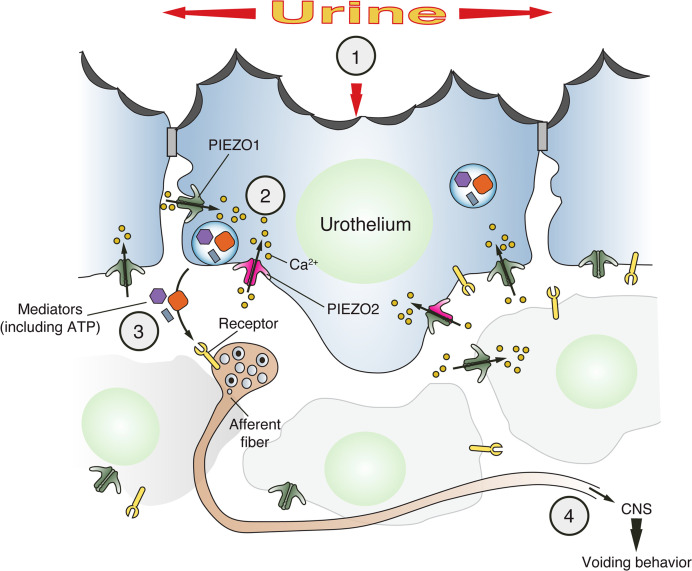

## Introduction

In addition to well-known senses that allow us to perceive external stimuli (e.g., taste, hearing, sight, touch, smell), body movement (vestibular senses), and body position (proprioception) is the “hidden” sense of interoception (1–3). It links the sensation of internal bodily functions, such as heartbeat, breathing, digestion, and bladder filling, to higher-order functions, including conscious awareness (e.g., my bladder is full), behavior (e.g., urination), and emotions (e.g., relief) (4). Integral to interoception are interoceptors, which are generally classified as sensory nerve endings (4, 5); however, other than astrocytes (6), a functional role for other non-neuronal tissues in interoception is an open question. In the case of the lower urinary tract (LUT), the stratified urothelium that lines most of these organs is thought to be a site of visceral sensation, and mechanotransduction in particular (7). For example, in response to bladder filling, the urothelium releases ATP, a mediator that is hypothesized to stimulate bladder activity and voiding function by communicating the degree of bladder fullness to subjacent tissues, including sensory afferents (7–9). Testing this “current model” in an in vivo setting has proved difficult because of a lack of fundamental insights into how tension in the plasma membrane of urothelial cells is sensed, how or if the associated mechanotransduction pathways contribute to mediator release from the serosal surfaces of the tissue, and whether these events are conducive to normal voiding function and behavior.

A variety of proteins instrumental to cellular mechanotransduction have been identified, including junction-associated proteins, G protein–coupled receptors, and stretch-activated channels (10–13). Nominally stretch-sensitive channels expressed by the urothelium include TRPV4, K^+^ channels, and the PIEZO channels (7). However, of this group, only PIEZO channels belong to the small class of channels (other members include TRAK1/2, KCNK2, and OSCA/TMEM63) that have been classified as bona fide mechanosensors, i.e., molecular force transducers that change their conformation in direct response to mechanical stimuli (12). Discovered by Ardem Patapoutian and colleagues using an siRNA screen (14), PIEZO1 (2521 amino acids in humans) and PIEZO2 (2752 amino acids in humans), form a small family of nonselective cation channels that are activated by various mechanical stimuli, including cell probing, changes in hydrostatic pressure, stretch, or laminar flow (14–17). Functional PIEZO channels can be reconstituted in vitro, their structures are known, and they regulate a diversity of stretch-sensitive processes in the body (18, 19).

Despite the rapid increase in knowledge of PIEZO channels over the past few years, our current understanding of PIEZO1 and PIEZO2 expression, distribution, and function in the urothelium is limited. The urothelium expresses *Piezo1* mRNA message, which reportedly exhibits circadian rhythmicity (20, 21). Using *Piezo1^tdT/tdT^* reporter mice, Dalghi et al. showed that the urothelium lining the renal pelvis, ureters, bladder, and urethra expresses PIEZO1, as do underlying interstitial fibroblasts, smooth muscle cells, endothelial cells, and mesothelial cells (22). In isolated urothelial cells, PIEZO1 along with TRPV4 are required for stretch-induced release of ATP (21), but whether release of mediators from native urothelium is also PIEZO1 dependent has not been reported. Currently, there are no insights into whether PIEZO1-dependent urothelial mechanotransduction pathways promote normal voiding function or behavior. While *Piezo2* expression is also reported in the urothelium (23), the expression and localization of PIEZO2 protein in the urinary tract is unknown. Intriguingly, patients deficient in *PIEZO2* expression exhibit voiding dysfunction (23), although this may not be the case for all patients (24). In addition, mouse LUT function is also reportedly dependent on *Piezo2* expression in sensory neurons and possibly the urothelium (23); however, whether *Piezo2* expression is critical for urothelial mechanotransduction, whether urothelial expressed PIEZO channels regulate voiding behavior, and whether PIEZO1 and PIEZO2 act in a coordinate fashion to regulate these events remain unknown.

To better understand roles for the urothelium in mechanotransduction and LUT interoception, we generated conditional urothelial *Piezo1*-knockout (KO), *Piezo2*-KO, and *Piezo1/2*-KO mice. Our studies reveal that the urothelium acts as a functional interoceptor, transducing mechanical stimuli in a PIEZO-dependent fashion, ultimately contributing to normal voiding function and behavior.

## Results

### Piezo1 and Piezo2 are expressed in mouse bladder urothelium.

**Figure 1:**
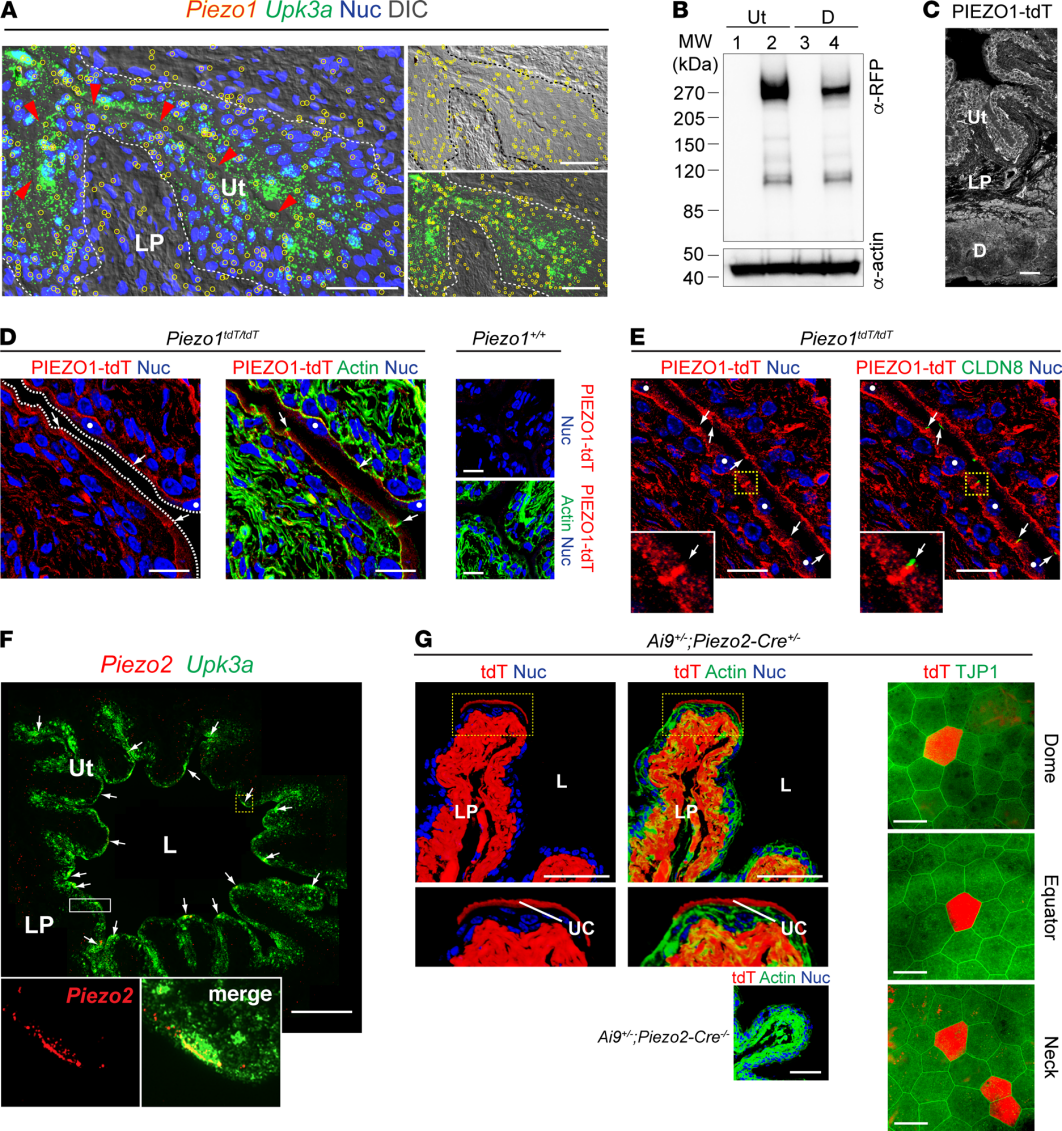
Expression and distribution of *Piezo1* and *Piezo2* in mouse bladder urothelium. (**A**) Distribution of *Piezo1* (signal dots in red, surrounded by yellow circles) and uroplakin 3A (*Upk3a*) (green) assessed using FISH. Urothelial boundary is outlined with white dashed lines (lumen indicated by red arrowheads), scale bars: 50 μm. (**B**) Western blot analysis of PIEZO1-tdT expression in urothelium (Ut) or detrusor (D) fractions taken from *Piezo1^+/+^* (lanes 1 and 3) or *Piezo1^tdT/tdT^* (lanes 2 and 4) mouse bladders. (**C**) Distribution of PIEZO1-tdT in bladder wall, scale bar: 100 μm. (**D** and **E**) Localization of PIEZO1-tdT with respect to the actin cytoskeleton or claudin-8 (CLDN8). Apical surface of umbrella cells are marked with white dashed lines, arrows point to the location of the junctional complex, closed white circles indicate umbrella cell nuclei, and the region of tissue in the yellow dashed box is magnified 2.9-fold (2.9×) in the insets. Scale bars: 20 μm. (**F**) *Piezo2* (red) and *Upk3a* (green) expression in mouse bladder urothelium defined using FISH. Arrows mark the position of *Piezo2*-expressing umbrella cells. The boxed region, indicated by a dashed yellow line, is magnified 14-fold (14×) in the insets. The larger panel is a photomerge of 11 images, collected using a wide-field microscope. The area bound by the rectangular box includes a stitching error when the samples were merged. Scale bar: 200 μm. (**G**) Expression of tdT in *Piezo2^Cre-IRES-GFP^* mice mated with Ai9 reporter mice. In the confocal images at the left and at the center, a single tdT-positive umbrella cell is located at the tip of a bladder rugae (also note tdT^+^ fibroblasts in LP). The region of the yellow dashed line is magnified 2-fold (2×) in the images below. Confocal images to the right show tdT-positive umbrella cells, viewed en face in whole-mounted bladder tissue. Examples of the regional expression of tdT-positive umbrella cells (and the tight junction protein TJP1) from the dome, equator, and neck region of the bladder are shown. All confocal images are 3D reconstructions of 32–48 optical sections. Scale bars: 100 μm. DIC, differential interference contrast; LP, lamina propria; L, lumen; Ut, urothelium.

Expression of *Piezo1* in mouse urothelial cells was initially assessed by fluorescence in situ hybridization (FISH), which confirmed *Piezo1* expression in all 3 layers of the urothelium, as well as subjacent cell types, including fibroblasts (Figure 1A). We used *Piezo1^tdT/tdT^* reporter mice (25), coupled with Western blot analysis, to show that PIEZO1–tandem-dimer Tomato (tdT) expression was enriched in a urothelial fraction (Figure 1B; urothelium to detrusor ratio of 1.00 ± 0.01 to 0.53 ± 0.10; normalized to urothelium, mean ± SEM, *n* = 3; see complete unedited blots in the supplemental material), which is consistent with the tissue distribution of PIEZO1-tdT assessed by immunofluorescence (Figure 1C). Furthermore, and like our previous report (7), we observed that PIEZO1-tdT was localized to the basal and lateral surfaces of umbrella cells, as well as at the plasma membranes of underlying intermediate and basal cells (Figure 1, D and E). PIEZO1 is reported to be enriched at the tight junction of intestinal adenocarcinoma-derived Caco2 cells (26); however, in native umbrella cells, PIEZO1-tdT was found along the lateral membrane but did not extend into the region of the CLDN8-labeled tight junction (Figure 1E).

Using FISH, we explored the expression of *Piezo2*, which was recently reported to be highly expressed in approximately 13% of umbrella cells but with up to 75% of umbrella cells expressing smaller amounts of message (23). To selectively label the urothelium, but not other bladder tissues, we used probes against *Upk3a*, which exhibits the highest expression in umbrella cells and lowest expression in basal cells (27–29). *Piezo2* message was mostly limited to a small population of apparently scattered umbrella cells, which also expressed relatively high amounts of *Upk3a* message (Figure 1F). Only small and variable amounts of *Piezo2* message were detected in the adjacent urothelial cells. *Piezo2* message was also observed in suburothelial fibroblasts and blood vessels. We attempted to immunolocalize PIEZO2 in the urothelium using commercial antibodies, or a previously published one (30), but were unable to convincingly confirm PIEZO2 expression in the bladder. We also employed a *Piezo2* mouse reporter line (*Piezo2^GFP-IRES-Cre^*), which expresses GFP fused to PIEZO2 as well as the Cre recombinase under the direction of the *Piezo2* promoter (30). However, we were unable to detect PIEZO2-GFP in any LUT tissue, even after amplifying the signal by use of anti-GFP antibodies.

We also mated *Piezo2^GFP-IRES-Cre^* mice with Ai9 reporter mice, which express tdT upon Cre-mediated recombination. This technique identifies sites of *Piezo2*-driven gene expression in adult tissues, but also serves as a form of lineage tracing, allowing for the identification of the adult progeny of *Piezo2*-expressing progenitor cells. The pattern of tdT expression was similar to that observed for FISH analysis: scattered tdT-positive umbrella cells, as well as tdT-positive interstitial fibroblasts (and blood vessels) (Figure 1G). The lack of expression in either basal or intermediate cells (both of which are precursors for umbrella cells) (31, 32) indicates that umbrella cells likely express *Piezo2* sometime after undergoing terminal differentiation. While the tdT-positive umbrella cells appeared to be randomly distributed, they formed a gradient along the dome-to-neck axis of the bladder, with increasing numbers near the neck region (Figure 1G). In sum, *Piezo1* message and PIEZO1 protein are expressed throughout the urothelium, whereas *Piezo2* message is mostly restricted to a scattered population of umbrella cells.

### Urothelial cells express Yoda1-responsive Piezo1 channels, but Piezo1-KO urothelium remains responsive to mechanical stimulation.

To understand whether PIEZO1 was integral to urothelial mechanotransduction, function, and interoception, we initially attempted to generate conditional urothelial *Piezo1*-KO mice by mating *Piezo1^fl/fl^* mice with those expressing inducible *Upk2*-*iCre^ERT2^* (33); however, this approach was ineffective at decreasing *Piezo1* expression by more than approximately 30%. We subsequently used a mouse strain with constitutive *Upk2*-driven *Cre* expression (*Upk2^Cre+/–^*) (34) and confirmed that Cre was expressed in all 3 layers of the urothelium lining the bladder, ureter, and proximal urethra but not in nonurothelial tissues (Supplemental Figure 1; supplemental material available online with this article; https://doi.org/10.1172/jci.insight.152984DS1). Using reverse transcription-quantitative PCR (RT-qPCR), we observed a significant approximately 75% decrease in *Piezo1* expression in urothelial lysates prepared from *Piezo1*-control (*Piezo1^fl/fl^ Upk2^Cre–/–^*) versus *Piezo1*-KO mice (*Piezo1^fl/fl^ Upk2^Cre+/–^*) (Supplemental Figure 2A). Because of the potential for contribution of *Piezo1* message from nonurothelial tissues in these lysates (e.g., from *Piezo1*-expressing fibroblasts), we also quantified *Piezo1* expression using FISH. In this case, we observed a significant approximately 85% reduction in urothelial *Piezo1* expression in *Piezo1*-KO mice versus controls (Supplemental Figure 2, B and C). Loss of *Piezo1* expression had no obvious effects on urothelial tissue morphology, expression of urothelial cell type–specific markers, or ultrastructure (Supplemental Figure 2, D and E).

**Figure 2:**
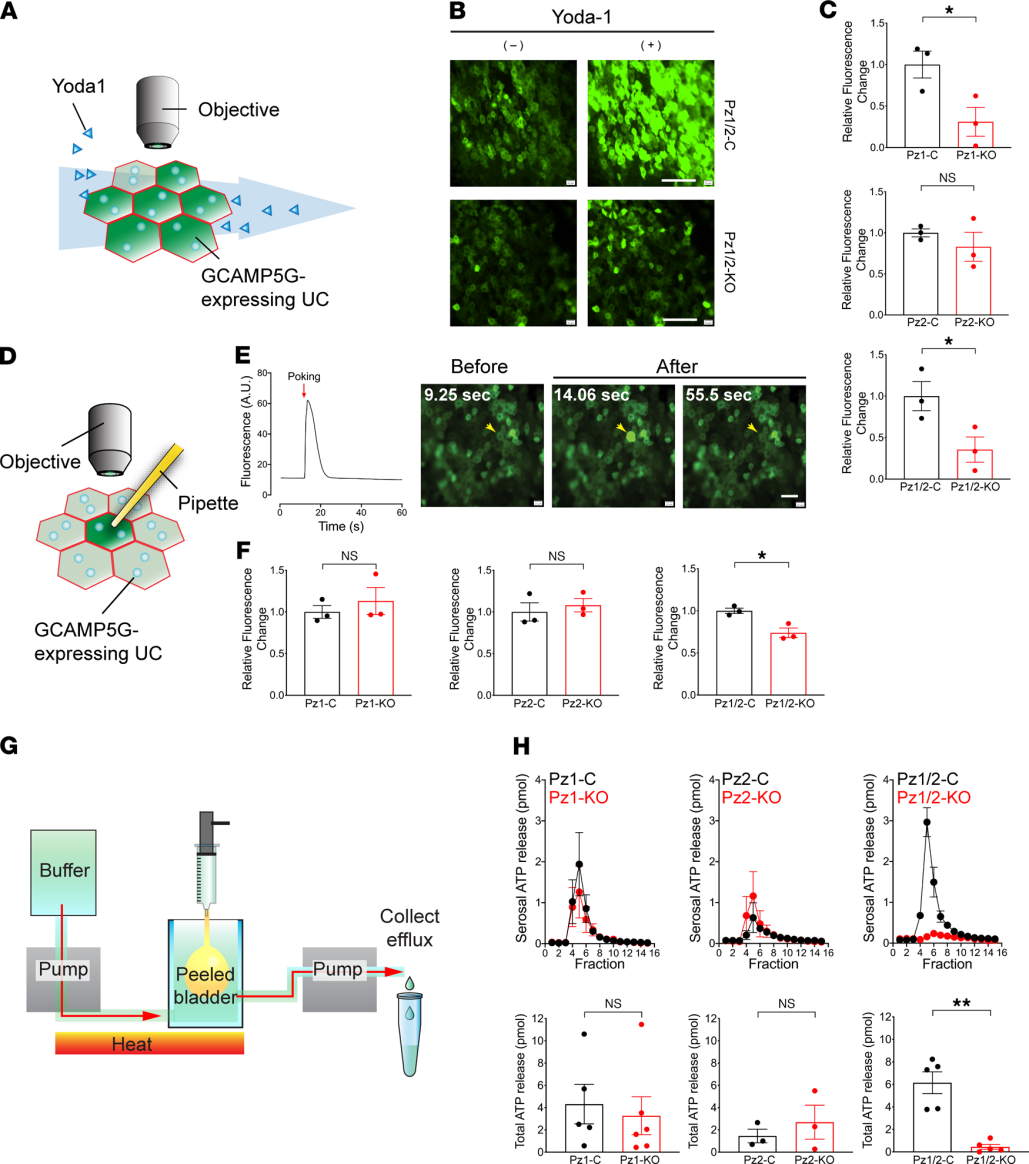
Evidence of PIEZO channel–dependent mechanotransduction in bladder urothelium. (**A**–**C**) Yoda1-stimulated PIEZO1 activation in GCAMP5G-transduced urothelium. (**A**) Diagram of experimental approach. (**B**) Example of Yoda1-induced [Ca^2+^]_i_ increases in *Piezo1/2*-control or *Piezo1/2*-KO urothelium. Scale bar: 100 μm. (**C**) Yoda1-induced changes in [Ca^2+^]_i_, normalized to control responses. Data are shown as mean ± SEM (*n* = 3). Data were analyzed using *t* tests and significant differences indicated with an asterisk (*P* ≤ 0.05). (**D**–**F**) Piezo channel dependence of poking-induced changes in [Ca^2+^]_i_. (**D**) Diagram depicting experimental approach. (**E**) Example of poking-induced increase in [Ca^2+^]_i_ in urothelium transduced with adenovirus encoding GCAMP5G. In the 3 images to the right, the indicated cell (yellow arrow) was poked at 10.0 seconds, and the changes in [Ca^2+^]_i_ were recorded over the next several seconds. Scale bar: 50 μm. (**F**) Poking-induced [Ca^2+^]_i_ changes in randomly selected umbrella cells. Data, normalized to matched controls, are shown as mean ± SEM (*n* = 3 animals for each group; the value of each animal is the average from 11–14 cells). Data were analyzed using *t* tests and significant differences indicated with an asterisk (*P* ≤ 0.05). (**G** and **H**) Dependence of serosal ATP release on *Piezo* expression. (**G**) Schematic of the experimental setup. (**H**) Upper panels: ATP release from peeled bladders of the indicated strain of mouse. Both males and females were used in this analysis. The peeled bladders were filled after fraction 3. Bottom panels: The total filling-induced ATP release from the serosal surfaces of peeled bladders was calculated. Data are shown as mean ± SEM (*Piezo1*-control, *n* = 5; *Piezo1*-KO, *n* = 6; *Piezo2*-control and *Piezo2*-KO, *n* = 3; *Piezo1*/2-control and *Piezo1/2*-KO, *n* = 5). Data were analyzed using *t* tests and significant differences indicated with a double asterisk (*P* ≤ 0.01).

The lack of access to the basolateral surfaces of native umbrella cells prevented us from measuring PIEZO channel activity using techniques like patch clamp. Instead, we measured changes in [Ca^2+^]_i_, a commonly used surrogate that increases downstream of PIEZO channel opening (14). We transduced the urothelium in situ with an adenovirus encoding the Ca^2+^ sensor GCAMP5G (35), made a sheet preparation of urothelium, and perfused it with the nominally PIEZO1-selective activator Yoda1 (Figure 2A) (36). This treatment stimulated a substantial increase in [Ca^2+^]_i_ (e.g., see Figure 2B), which was significantly reduced in urothelial sheets prepared from conditional urothelial *Piezo1*-KO mice, but not in urothelium derived from *Piezo2*-KO mice (Figure 2C). Unfortunately, there are no known agonists of PIEZO2 channels, and thus we could not use a similar strategy to confirm the presence of functional PIEZO2 channels in the urothelium.

To assess the sensitivity of urothelial PIEZO1 channels to mechanical stimuli, we used fire-polished and sealed patch pipettes to gently “poke” random umbrella cells (Figure 2D). This stimulus elicits large changes in PIEZO channel activity, and in the case of PIEZO1, poking is approximately 20-fold better than increasing membrane tension by use of suction (14, 30). Poking resulted in an instantaneous rise in umbrella cell [Ca^2+^]_i_, followed by a rapid fall (Figure 2E and Supplemental Video 1). The reversibility of the effect, combined with the ability to trigger repeated [Ca^2+^]_i_ spikes from the same cell, confirms that the cells were not damaged by this treatment. A careful examination of the videos revealed that adjacent cells, including those several cell lengths from the target cell, exhibited [Ca^2+^]_i_ responses but after a several-second delay (Supplemental Video 1). The nature of these secondary events was not explored further. Surprisingly, we did not detect a significant difference in poking-induced [Ca^2+^]_i_ responses when comparing *Piezo1*-control umbrella cells to those from *Piezo1*-KO tissue (Figure 2F and Supplemental Figure 3). While it is feasible that mechanical poking is ineffective at stimulating urothelial expressed PIEZO1 channels, an alternative possibility is that the loss of *Piezo1* is compensated by expression of *Piezo2* or other gene products. This possibility is explored in more detail below.

### Serosal release of ATP from the native urothelium is not impaired in Piezo1-KO mice.

The urothelium is proposed to regulate LUT function by releasing mediators, ATP in particular, in response to mechanical stimuli such as bladder filling (7, 37). To test the *Piezo1* dependence of ATP release from native urothelium, we used a “peeled” bladder preparation (Figure 2G and Supplemental Figure 4), which allowed us to measure release of ATP from the serosal surfaces of the urothelium and in response to filling (albeit from the urethral side). We previously showed, using dissected rabbit urothelium, that the urothelium and not the underlying fibroblasts are the primary source of ATP (38). The urothelium in these preparations was intact and maintained tight junctions, while the majority of lamina propria, muscularis, and serosa was removed (Supplemental Figure 4). We observed that ATP was released from the serosal surfaces of both *Piezo1*-control and *Piezo1*-KO mice in peeled bladders (Figure 2H). No significant differences were observed between these 2 groups. Thus, unlike the previous report that ATP release from isolated urothelial cells is (partially) *Piezo1* dependent (21), serosal release of ATP from the native urothelium was not significantly affected by loss of *Piezo1* expression.

### Piezo1-KO mice exhibit near-normal voiding function and voiding behavior.

**Figure 3:**
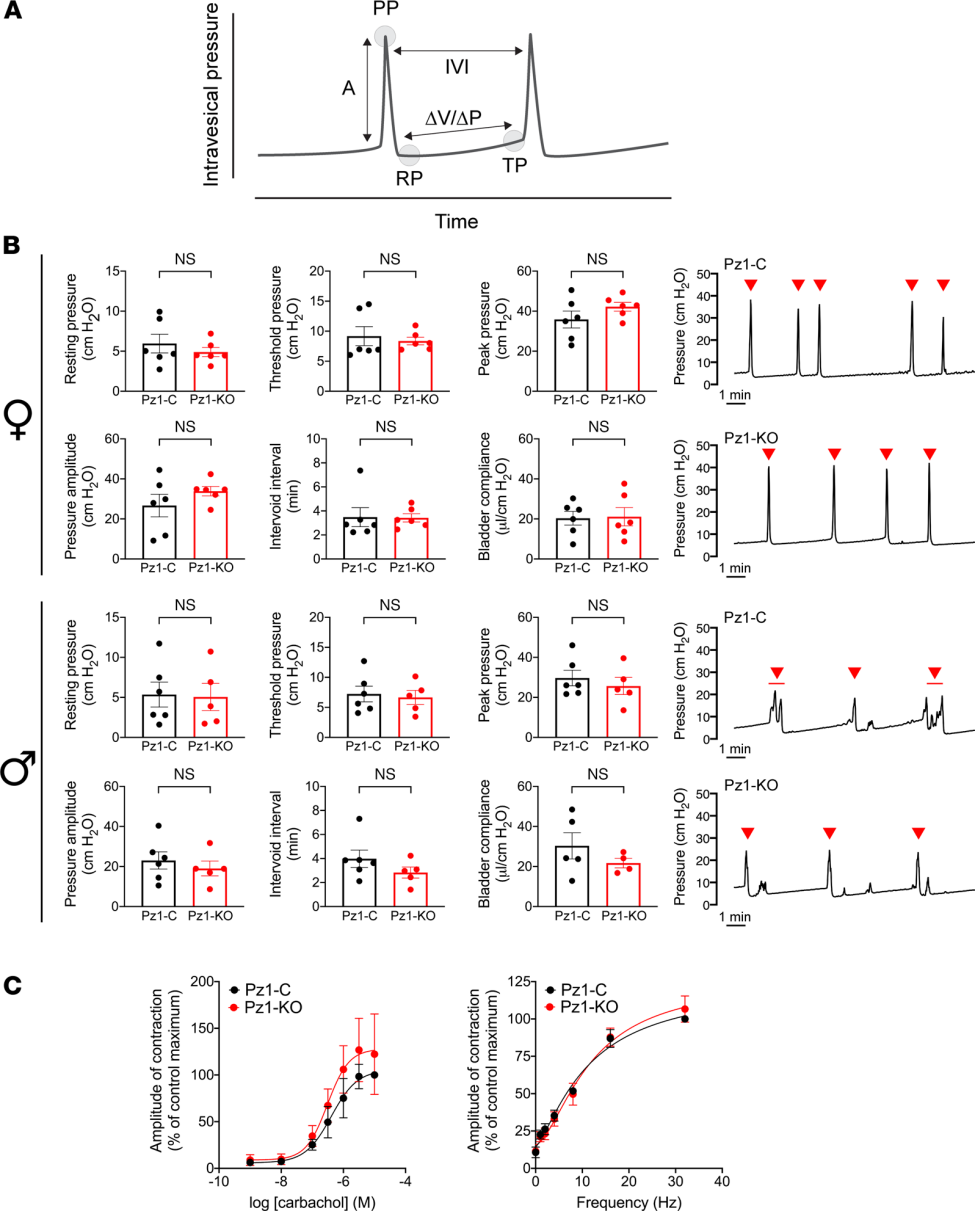
Bladder function in *Piezo1*-control and *Piezo1*-KO mice. (**A**) Example cystometrogram. A, amplitude (PP-TP); IVI, intervoid interval (time between voiding events); PP, peak pressure (associated with voiding event); RP, resting pressure; TP, threshold pressure; ΔV/ΔP (compliance), change in pressure in response to an incremental change in volume. (**B**) Comparison of cystometric parameters for male and female, urethane-anesthetized *Piezo1*-control and *Piezo1*-KO mice. Voiding events are marked with red arrowheads (line below arrowhead indicates a single voiding event with multiple pressure spikes). Data, analyzed using Mann-Whitney tests, are shown as mean ± SEM (*Piezo1*-control, males and females, *n* = 6; *Piezo1*-KO female, *n* = 6; *Piezo1*-KO male, *n* = 5). (**C**) Contraction of muscle strips in response to carbachol and electric field stimulation. Data are shown as mean ± SEM (*n* = 3).

To assess LUT function, we performed continuous cystometry in urethane-anesthetized *Piezo1*-control and *Piezo1*-KO mice. In female mice, none of the measured cystometric parameters were significantly affected by loss of urothelial *Piezo1* expression (Figure 3, A and B). This included voiding efficiency (1.26 ± 0.08 in *Piezo1*-control mice versus 1.17 ± 0.15 in *Piezo1*-KO mice; no significant difference, 2-tailed *t* test), a measure of the ability of the bladder to completely release its contents because of efficient detrusor contractions coupled with coordinate “opening” of the bladder sphincter. Relative to *Piezo1*-control mice, male *Piezo1*-KO mice also exhibited no significant differences in their cystometric parameters (Figure 3B). As it is difficult to accurately collect fluid from male mice, we were not able to measure their bladder efficiency. To further assess detrusor function in *Piezo1*-KO mice, we measured contractions of isolated, full-thickness bladder strips. However, there were no significant effects, even after triggering strong detrusor contractions using carbachol treatment or electrical field stimulation (Figure 3C).

**Figure 4:**
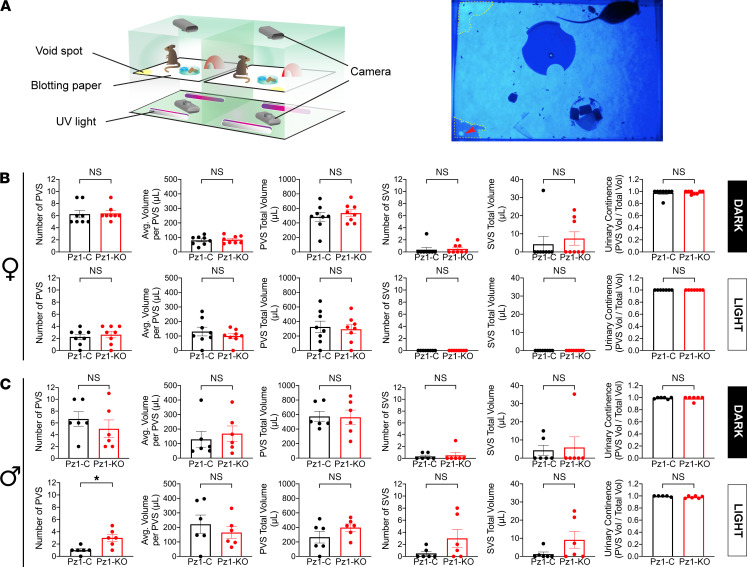
Voiding behavior in freely mobile *Piezo1*-control and *Piezo1*-KO mice. (**A**) Diagram of void spot chamber. Right panel: Representative video still of a mouse in a void spot chamber, illuminated from below with UV light, and recorded using the top camera. Primary void spots (PVSs) are outlined with dashed yellow lines. A secondary void spot (SVS), on top of a previous PVS, is indicated with a red arrowhead. (**B** and **C**) Void spot parameters in female (**B**) and male (**C**) mice analyzed over a 6-hour time window during the dark phase or light phase. Data are shown as mean ± SEM (*Piezo1*-control and *Piezo1*-KO females, *n* = 8; *Piezo1*-control and *Piezo1*-KO males, *n* = 6). Data were analyzed using a Mann-Whitney test and significant differences indicated with a single asterisk (*P* ≤ 0.05).

Finally, we performed video-monitored void spot assays on *Piezo1*-control and *Piezo1*-KO mice. Unlike cystometry, these assays were performed on freely moving, nonanesthetized mice, allowing us to assess conscious voiding behavior (Figure 4A). Because *Piezo1* expression is reported to exhibit circadian rhythmicity, with 4-fold greater expression during the mouse’s active dark phase (20, 39), we measured voiding behavior in a 6-hour time window during either the light or dark phase. We also assessed whether there were any sex-related differences. As in previous studies, we sorted urine output into 2 classes: PVSs (>20 μL) or SVSs (those <20 μL) (40–42). While the latter are uncommon in control mice (except in the case of aggressive alpha males), they increase in animals exhibiting urinary incontinence (i.e., leakage), including mice with experimental cystitis, mice with outlet obstruction, or those exposed to stress (43–46).

Female *Piezo1*-control mice had approximately 1 PVS per hour during their active dark phase, but approximately one-third of that number was observed during the light phase, when the mice were most often inactive (i.e, resting or sleeping; Figure 4B). The relatively low numbers of PVSs during the light phase, the lack of SVSs, and the increase in PVSs during the dark phase confirm that the mice retained their circadian patterns of urination and were not overtly stressed by being monitored. When comparing female *Piezo1*-control mice to *Piezo1*-KO ones, we noted no significant differences in the PVS or SVS parameters (Figure 4B). Technically, the average volume/PVS is indeterminate when the number of PVSs falls to 0, which we observed in a few animals. However, if we performed a secondary analysis in which we excluded those animals with no voids, the values for this and other parameters remained nonsignificant. We also calculated a urinary continence score; however, we noted no significant effects on continence (Figure 4B). Again, when the PVS is 0, the continence score becomes indeterminate. In this case, the values were excluded, as a value of 0 would indicate complete incontinence, which is the opposite of not voiding. Like females, *Piezo1*-control male mice had larger numbers of PVSs during their dark phase compared with their light phase, and few SVSs were observed. During the dark phase, none of the measured parameters were significantly different between *Piezo1*-control and *Piezo1*-KO male mice (Figure 4C). However, we observed a significant increase in number of PVSs during the inactive light phase in male *Piezo1*-KO mice (independent of including or excluding animals that did not void; Figure 4C).

In summary, poking-induced changes in [Ca^2+^]_i_, serosal ATP release, cystometry, and voiding function were largely unaffected in *Piezo1*-KO mice. However, and opposite of the predictions of the current model, *Piezo1*-KO male mice exhibited a form of “nocturia,” in which they voided more frequently during their less active light phase.

### Conditional urothelial Piezo2-KO mice display normal mechanotransduction and LUT function, but male mice exhibit defects in voiding behavior.

It was recently reported that conditional urothelial *Piezo2*-KO mice have abnormalities in bladder function (assessed using cystometry) and sphincter function (23). This study did not, however, assess whether urothelial mechanotransduction or voiding behavior are affected in *Piezo2*-KO animals. To explore these possibilities, we generated conditional urothelial *Piezo2*-control and *Piezo2*-KO mice. We were unable to quantify any decrease in *Piezo2* expression using RT-qPCR because the amount of *Piezo2* expression in the urothelium of *Piezo2*-control mice was too low to accurately measure by this technique. However, by using FISH to estimate the number of *Piezo2*-expressing umbrella cells per length of luminal membrane, we confirmed that *Piezo2* expression was reduced to almost 0 in *Piezo2*-KO mice (Supplemental Figure 5A). There was no significant difference between *Piezo2*-control and *Piezo2*-KO responses to poking-induced changes in [Ca^2+^]_i_ (Figure 2C). Moreover, robust response to Yoda1 indicated that umbrella cells lacking *Piezo2* expression still expressed functional PIEZO1 channels (Figure 2C). We could not demonstrate any reliance of serosal ATP release on *Piezo2* (Figure 2H), and in cystometry, we could not detect significant differences in bladder function between *Piezo2*-control versus *Piezo2*-KO male or female mice (Supplemental Figure 6). There was no significant effect on female *Piezo2*-KO voiding efficiency (1.03 ± 0.10 in *Piezo2*-control mice versus 1.12 ± 0.03 in *Piezo2*-KO mice; 2-tailed *t* test).

**Figure 5:**
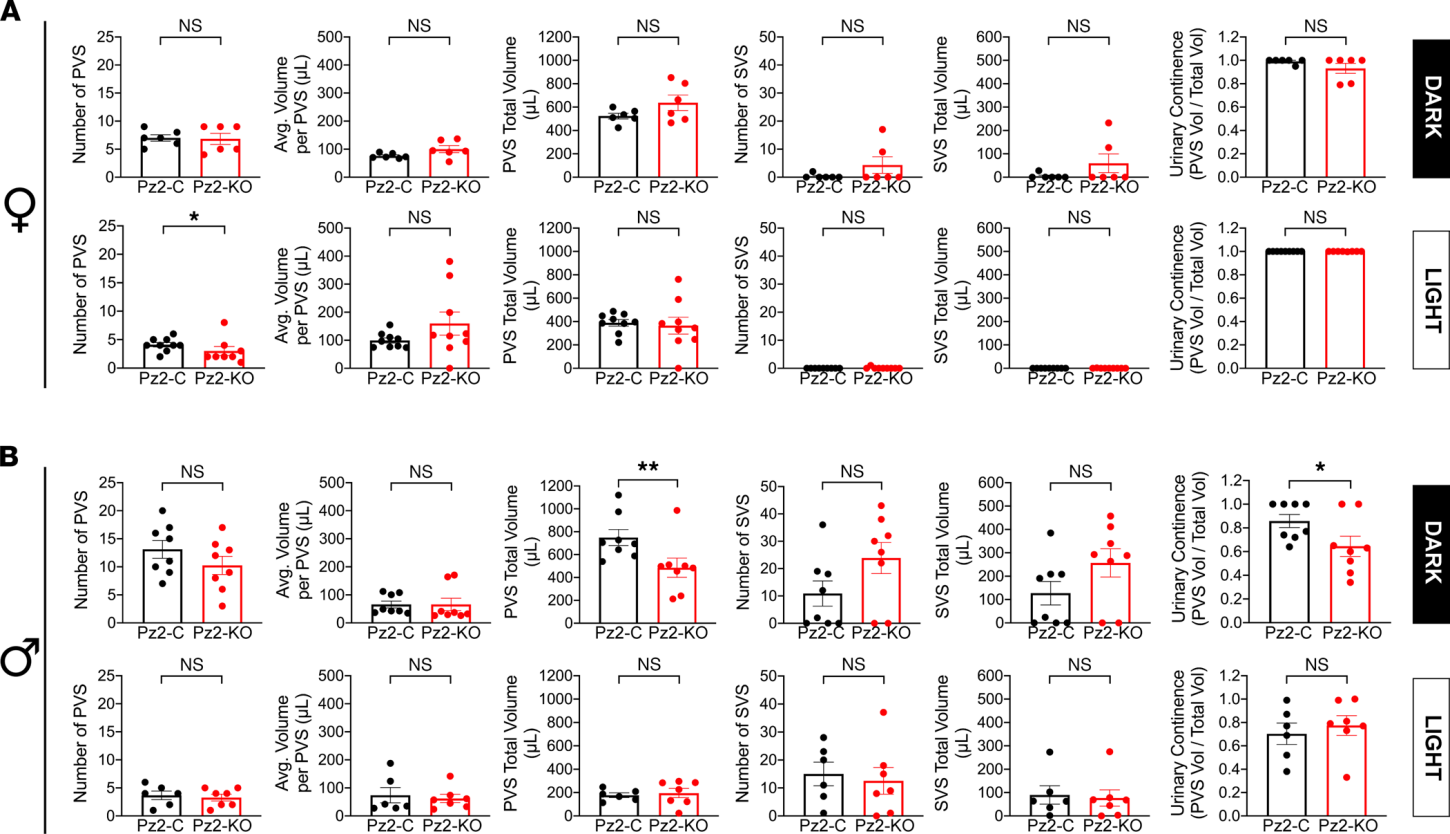
Voiding behavior in freely mobile *Piezo2*-control and *Piezo2*-KO mice. (**A** and **B**) Void spot parameters in female (**A**) and male (**B**) mice were analyzed over a 6-hour time window during the dark phase or light phase. Data are shown as mean ± SEM (dark-phase females, *Piezo2*-control and *Piezo2*-KO, *n* = 6; light-phase females, *Piezo2*-control and *Piezo2*-KO, *n* = 9; dark-phase males, *Piezo2*-control and *Piezo2*-KO, *n* = 8; light-phase males, *Piezo2*-control, *n* = 6, *Piezo2*-KO, *n* = 7). Data were analyzed using a Mann-Whitney test and significant differences indicated with a single asterisk (*P* ≤ 0.05) or a double asterisk (*P* ≤ 0.01).

Finally, we assessed voiding behavior in *Piezo2*-control versus *Piezo2*-KO mice (Figure 5A). In the case of female mice, we noted a significant decrease in the number of PVSs during the light phase, but this did not affect PVS total volume. When a secondary statistical analysis was performed, in which we excluded the animal that did not void, the differences became not significant. The most striking change we observed was in male mice during their active dark phase. Here, we noted a decrease in continence, which apparently resulted from a significant decrease in total PVS volume (Figure 5B). No significant differences were observed for the other measured parameters or during the male light phase. In sum, conditional urothelial male *Piezo2*-KO mice had a reduced total PVS volume in their dark phase, likely contributing to the overall decrease in continence. Interestingly, this occurred in the absence of any *Piezo2*-KO–dependent change in ATP release or in sensitivity to mechanical stimulation.

### Role for urothelial mechanotransduction is revealed in Piezo1/2-KO mice.

Up to this point, it was possible to conclude that urothelial expressed PIEZO channels have limited (or no) roles in urothelial mechanotransduction or voiding function. However, the relatedness of PIEZO1 and PIEZO2 led us to determine if loss of urothelial *Piezo1* expression was compensated for by upregulated expression of *Piezo2*. However, we could not detect a significant difference in urothelial *Piezo2* expression in *Piezo1*-control versus *Piezo1*-KO mice (1.00 ± 0.12 and 0.95 ± 0.14, respectively; data normalized to *Piezo1*-control values, mean ± SEM, *n* = 3; 2-tailed *t* test). To determine whether expression of both channels was necessary, we generated *Piezo1/2*-control or *Piezo1/2*-KO mice. FISH was used to confirm that *Piezo1/2*-KO mice had significantly lower amounts of *Piezo1* and *Piezo2* expression compared with *Piezo1/2*-control mice (Supplemental Figure 7). Furthermore, *Piezo1/2*-KO umbrella cells were also significantly less responsive to treatment with Yoda1 (Figure 2C), confirming a significant reduction in their expression of PIEZO1. The urothelium of *Piezo1/2*-KO mice exhibited no obvious abnormalities, maintaining a WT like organization and expression of urothelial marker proteins (Supplemental Figure 8).

We first determined if the *Piezo1/2*-KO mice would reveal the existence of PIEZO1/2-dependent mechanotransduction pathways in the urothelium. Consistent with this possibility, *Piezo1/2*-KO umbrella cells exhibited a significant reduction in poking-induced changes in [Ca^2+^]_i_, (Figure 2F). The mechanotransduction pathway that contributes to the remaining poking-induced rise in [Ca^2+^]_i_ is unknown and was not explored further. Next, we measured ATP release from the urothelium of *Piezo1/2*-control and *Piezo1/2*-KO peeled bladders. Strikingly, we observed that serosal ATP release was blocked in peeled *Piezo1/2*-KO bladders (Figure 2G), revealing an almost complete dependence of serosal ATP release on PIEZO1/2 channel–dependent mechanotransduction.

**Figure 6:**
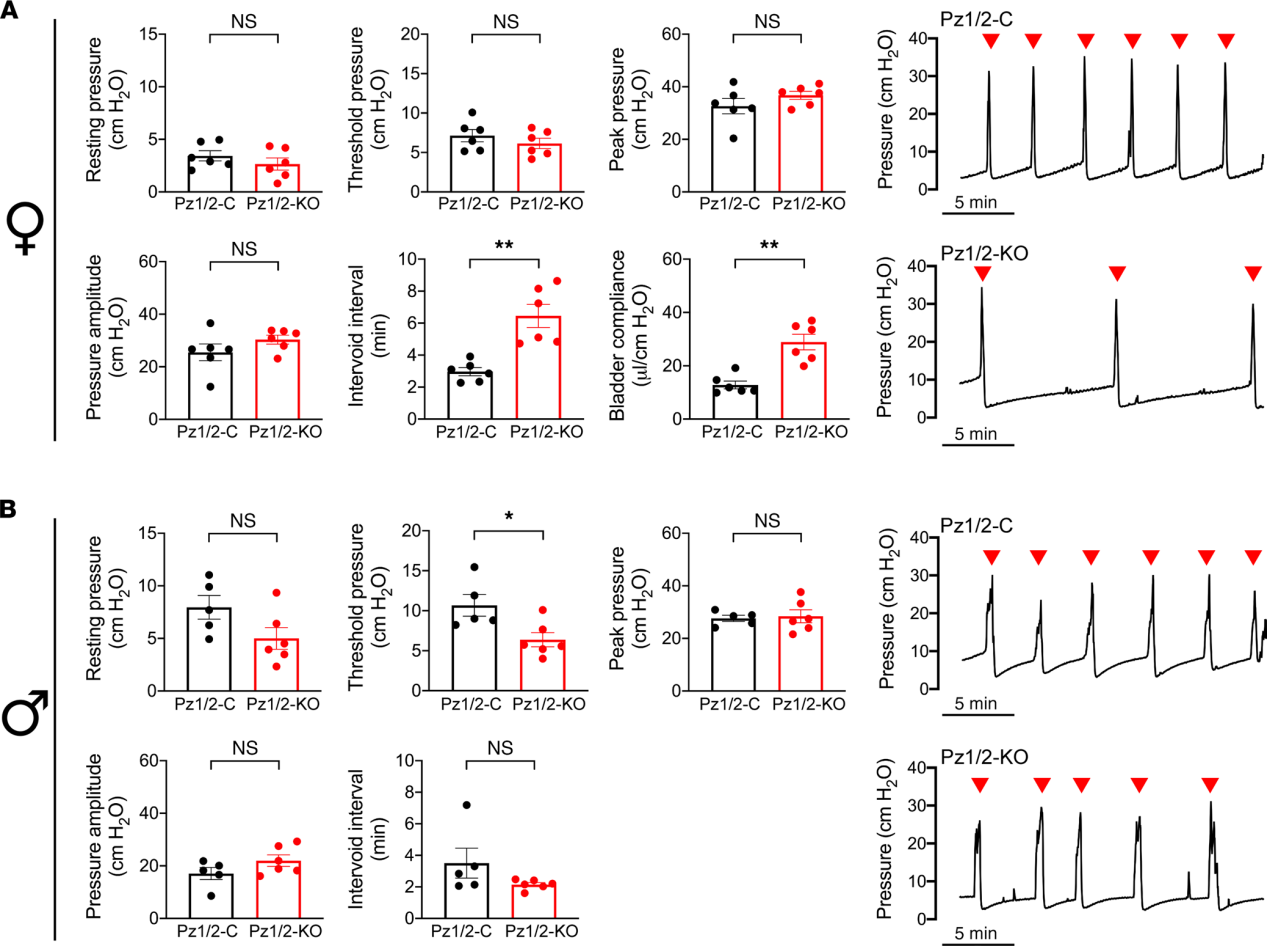
Voiding function of anesthetized *Piezo1/2*-control and *Piezo1/2*-KO mice as assessed by cystometry. (**A** and **B**) Urethane-anesthetized female (**A**) or male (**B**) mice were subjected to cystometry. Representative cystometrograms are shown to the right of the figure. Data are shown as mean ± SEM (female *Piezo1/2*-control and *Piezo1/2*-KO, *n* = 6; male *Piezo1/2*-control, *n* = 5; male *Piezo1/2*-KO, *n* = 6). Data were analyzed using Mann-Whitney tests and significant differences indicated with a single asterisk (*P* ≤ 0.05) or a double one (*P* ≤ 0.01).

The dramatic loss of serosal ATP release in Piezo1/2-KO mouse urothelium allowed us to assess whether urothelial ATP release (and possibly other mediators) is integral to voiding function. In the case of female *Piezo1/2*-KO mice, we observed no significant effects on resting pressure, pressure threshold, maximum contraction, or amplitude in animals undergoing cystometry (Figure 6A), nor was there any significant difference in voiding efficiency (1.09 ± 0.02 in *Piezo1/2*-control and 1.02 ± 0.05 in *Piezo1/2*-KO mice; data normalized to *Piezo1/2*-control, mean ± SEM, *n* = 6, data not significantly different using Mann-Whitney *U* test). Instead, we noted a significant increase in intervoid interval and an associated increase in bladder compliance (Figure 6A). This would indicate that anesthetized female *Piezo1/2*-KO mice were less sensitive to bladder filling, the bladder required more volume to initiate a contraction, and the detrusor of *Piezo1/2*-KO mice was likely more relaxed. However, in the case of male *Piezo1/2*-KO mice, there was no effect on intervoid interval or compliance. Instead, their threshold pressure was significantly decreased relative to *Piezo1/2*-control mice (Figure 6B), indicating that the pressure needed to trigger voiding was reduced in these mice. None of the other cystometric parameters were significantly affected.

**Figure 7:**
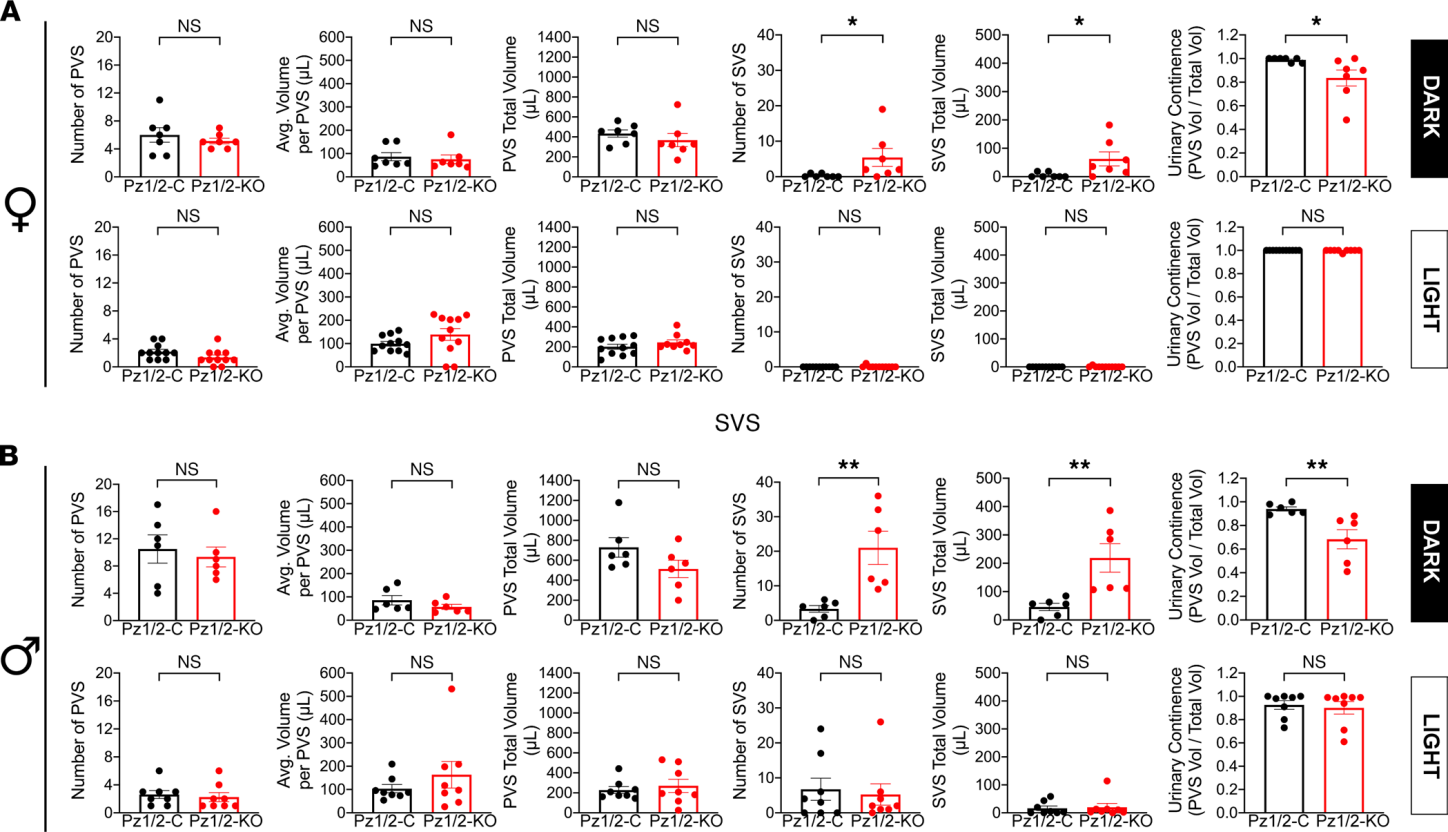
Voiding behavior in freely mobile *Piezo1/2*-control or *Piezo1/2*-KO mice. (**A** and **B**) Void spot parameters in female (**A**) and male (**B**) mice were analyzed in a 6-hour time window during the dark phase or light phase. Data are shown as mean ± SEM (dark-phase females, *Piezo1/2*-control and *Piezo1/2*-KO, *n* = 7; light-phase females, *Piezo1/2*-control and *Piezo1/2*-KO, *n* = 11; dark-phase males, *Piezo1/2*-control and *Piezo1/2*-KO, *n* = 6; light-phase males, *Piezo1/2*-control and *Piezo1/2*-KO, *n* = 8). Data were analyzed using Mann-Whitney tests and significant differences indicated with a single asterisk (*P* ≤ 0.05) or with a double asterisk (*P* ≤ 0.01).

Finally, we assessed voiding behavior in the *Piezo1/2*-KO mice. Despite the cystometry results described above, freely mobile unanesthetized *Piezo1/2*-KO female mice exhibited no signs of PVS or SVS defects during their inactive light phase (Figure 7A). However, if we performed a secondary analysis where we excluded nonvoiders, the average volume/PVS was significantly different between *Piezo1/2*-control versus *Piezo1/2*-KO mice. Somewhat like the *Piezo2*-KO male mice described above (Figure 5B), we observed a significant decrease in bladder continence in *Piezo1/2*-KO female mice; however, the latter exhibited no PVS defects but instead presented with an increase in SVS number and total SVS volume (Figure 7A). We note that SVSs were individual events, not obviously tied in time to PVS, and were voluntary in the sense that animals moved to the corners, urinated, and then left. In the case of male *Piezo1/2*-KO mice, we observed no significant differences in PVS number, average volume per PVS, or total PVS volume during their dark phase, but like female mice they did present with a significant increase in SVS number and total SVS volume (Figure 7B). In turn, this led to a significant decrease in continence. No significant differences in voiding activity were observed during the light phase of male *Piezo1/2*-KO mice versus *Piezo1/2*-control ones (Figure 7B). Taken together, our studies reveal an important role for PIEZO1/2 channels in urothelial mechanotransduction, including functions in mechanosensing, serosal release of ATP, and modulation of voiding function and behavior.

## Discussion

Visceral organs experience mechanical forces that include rhythmic contractions (e.g., heartbeat and peristalsis), and in most cases gases, fluids, and solids enter; exit; or flow through them. While PIEZO channels are important to visceral organ functions that include blood pressure sensing (47), lung inflation (48), and release of hormones (49), few studies have made direct links between Piezo channel–dependent mechanotransduction and behavior, a critical link that underpins interoception. Perhaps the clearest evidence to date is the coupling of Piezo expression to neurons that innervate the *Drosophila melanogaster* crop and anterior midgut and are important for regulating fly feeding behavior (50, 51). Other potential examples include voiding anomalies in patients with *PIEZO2* mutations or in *Hoxb8-cre* *Piezo2^fl/fl^* mice (23) that also suffer from severe defects in motility and proprioception (52). However, the reported expression of *Hoxb8* in all mouse bladder tissue types (28), combined with the reported expression of *Piezo2* in blood vessels, fibroblasts, neurons, and urothelium, makes it difficult to parse which tissue(s) is acting as the interoceptor (this manuscript; refs. 23, 25, 52). Marshall et al. also reported that *Piezo2* deletion in afferent nerves or the urothelium alters LUT function (23); however, they did not assess if urothelial mechanotransduction was affected in these *Piezo2*-KO mice, nor did they measure voiding behavior in these mice. Using conditional urothelial *Piezo*-KO mice, our studies reveal the following insights: (i) urothelial mechanotransduction depends in part on expression of *Piezo1* or *Piezo2*; (ii) the urothelium acts as a mechanotransducer, and ATP release from the serosal surfaces of the native urothelium is *Piezo1/2* dependent; and (iii) the urothelium functions as a non-neuronal interoceptor, linking PIEZO1/2 mechanotransduction to serosal mediator release (i.e., ATP) and to normal voiding function and behavior.

Our initial insight is that expression of *Piezo1* or *Piezo2* is integral to umbrella cell responses to mechanical stimulation. Interestingly, gentle poking of randomly chosen umbrella cells increases [Ca^2+^]_i_, but only in *Piezo1/2*-KO mice did we observe an inhibition of these responses. This dual PIEZO channel requirement is surprising given that PIEZO1 is apparently expressed in all urothelial cells (and could be stimulated by Yoda1), whereas *Piezo2* expression is seemingly limited to a small population of scattered umbrella cells, whose function remains unknown. Unfortunately, we still have no insights into the expression, distribution, and rate of turnover of the PIEZO2 protein due to lack of suitable reagents. The most parsimonious solution to our conundrum would be that loss of *Piezo1* would be compensated for by an increase in *Piezo2* expression; however, we found no evidence that this was occurring. An additional possibility is that the PIEZO1/2-expressing umbrella cells produce a diffusible signaling molecule that is critical for mechanical responses in adjacent urothelial cells. This hypothesized signaling molecule could function by way of a paracrine signaling pathway, by way of connexin channels that are expressed by the urothelium (20, 53). In this model, our results could be explained, for example, if PIEZO1 and PIEZO2 form heteromeric channels, but in the absence of its binding partner, the PIEZO channels form homomeric ones that still retain some degree of functionality. An alternative possibility relates to our use of mouse strains that constitutively express *Upk2*-driven Cre, a necessity to effectively reduce *Piezo1/2* expression. As a result, loss of *Piezo1* alone or *Piezo2* alone could be compensated for during development (and/or postnatally) by altered expression of other genes integral to the mechanotransduction pathway. In this scenario, loss of both *Piezo* channel genes becomes too much to overcome. Finally, we also observed a large fraction of poking-induced [Ca^2+^]_i_ that was not attenuated in *Piezo1/2*-KO mice, indicating that other mechanotransduction pathways are likely operating in the urothelium. The nature of these pathways remains mysterious, but the urothelium potentially expresses multiple stretch-activated channel activities (reviewed in ref. 7). For example, we have described a nonselective, stretch-activated channel activity at the apical surfaces of umbrella cells (54), and the basolaterally distributed TRPV4 channel was previously implicated in regulating bladder function and in stretch-induced [Ca^2+^]_i_ changes in isolated urothelial cells (21, 55).

Our second insight relates to the PIEZO channel dependence of serosal mediator release from the native urothelium and its hypothesized role in communication with subjacent tissues (37). In the case of ATP, a plethora of studies have explored the mechanisms and pathways of urothelial ATP release, including reports that filling-induced ATP release is decreased in *P2rx3*-, *Trpv1*-, *Trpv4*-, *Slc17a9*- (VNUT), and *Cnx43*-KO mice (53, 55–59) or increased in urothelial conditional β_1_ integrin–KO (*Itgb1*-KO) mice (60). However, none of these studies unambiguously identified the mechanosensor, and in all cases, only mucosal ATP release was measured. While mucosal ATP can trigger events via a transmural pathway (7, 61), it is most likely that serosally released mediators are integral to urothelial interoception. Miyamoto et al. reported that stretch-induced ATP release from isolated mouse urothelial cells is (partially) *Piezo1* dependent (21), and subsequent studies showed the *Piezo1* dependence of endothelial cell ATP release (62). Using a peeled bladder preparation, we demonstrate that filling-induced release of ATP from the serosal surface of the native urothelium is wholly dependent on a PIEZO1/2 mechanotransduction pathway. While this preparation is arguably more physiologically relevant than isolated cells, by necessity it requires the removal of blood vessels, fibroblasts, nerves, and smooth muscle cells, whose contributions may include effector functions (e.g., release of mediators), modulation of urothelial signaling, and roles in defining the mechanical properties of the mucosa. Despite these limitations, the tools we have employed make it possible to begin exploring whether the serosal (and mucosal) release of other urothelial mediators (e.g., acetylcholine, adenosine, prostaglandins, NO) (7) is similarly PIEZO1/2 dependent.

Our final insight relates to the hypothesized role that the urothelium plays in regulating LUT function and voiding behavior, and in particular its role as an interoceptor. In the current model, bladder filling causes the urothelium to release ATP, which is hypothesized to act in a stimulatory manner to promote voiding function downstream of a local urothelial/afferent reflex (or via effects on other subepithelial tissues) (63). Notwithstanding the possibility that other mediators are also affected, the almost complete loss of serosal ATP release in *Piezo1/2*-KO mice afforded us an ideal opportunity to test this model. Even so, the phenotype of the *Piezo1/2*-KO mice varied depending on whether we measured voluntary voiding behavior (i.e., void spot assays) versus involuntary reflex micturition (i.e., cystometry), on the animal sex, and on the light phase during which measurements were made. The extended intercontraction interval and increased compliance we observed in female *Piezo1/2*-KO undergoing cystometry are consistent with the current model, as is the increased average volume per PVS during the light phase of conscious female mice (when we exclude nonvoiding events). However, the decreased threshold pressure that we measured when performing cystometry in *Piezo1/2*-KO male mice would not be, nor would the observation that voiding behavior during the light phase of male *Piezo1/2*-KO mice was not significantly different than control mice. The greatest divergence from the current model occurred when we assessed the voiding behavior of *Piezo1/2*-KO mice during their active dark phase. In both sexes, we observed a marked increase in SVSs (but no significant effect on PVSs), indicating urinary incontinence.

The proximal cause of this incontinence is unknown, but one possibility is that during the dark phase, urothelial released ATP functions in an inhibitory fashion to dampen pathways that give rise to urinary incontinence. For example, the urethral sphincter is under control of a guarding reflex that strengthens urethral tone as the bladder becomes full (64). If urothelial ATP regulates these pathways, then loss of this mediator could allow urine leakage. Alternatively, incontinence may result from increased (or decreased) production of other mediators that are also under the control of the urothelial PIEZO channel–dependent mechanotransduction pathway. This could explain why *Piezo1*-KO and *Piezo2*-KO mice, males in particular, exhibited alterations in voiding behavior (nocturia and incontinence, respectively), even though serosal ATP release was apparently unaffected in these animals. In any case, both the mediators involved, and the target tissues (e.g., afferent nerve endings, detrusor, or urethral outlet) affected, will need to be defined by future studies. A final alternative is related to original identification of PIEZO1 (known as FAM38A at the time) as a regulator of ITGB1 activity and cell motility (65, 66). Intriguingly, conditional urothelial *Itgb1*-KO mice are reported to exhibit incontinence, producing increased numbers of small void spots (0.8–4.0 μL range) (60). While the *Itgb1*-KO mouse phenotype does not completely phenocopy that of *Piezo1/2*-KO mice (e.g., *Itgb1*-KO mice have large numbers of nonvoiding contractions), integrins are known to play important roles in mechanotransduction (67), and thus some aspects of the incontinence we observe may involve the interplay between PIEZO channel–dependent signaling and that associated with ITGB1.

Finally, although urination can bring a feeling of relief in a normal setting, in pathological settings, LUT symptoms (including urgency, frequency, hesitancy, and nocturia) and incontinence give rise to emotional distress. As noted above, a subset of patients with *PIEZO2* mutations exhibit LUT dysfunction (23). Their symptoms include bladder underactivity, or urgency in some cases, and stress urinary incontinence. However, and unlike our urothelium-focused studies, patients with *PIEZO2* mutations are deficient in *PIEZO2* expression in all tissues, including the urothelium and nervous system, and are more closely mimicked by the aforementioned *Hoxb8-cre* *Piezo2^fl/fl^* mouse, which are also described as being incontinent (23). Other mouse studies have proposed (although not directly tested) roles for *Piezo1* in partial bladder outlet obstruction, cystitis, and conditions that may arise from altered circadian expression of genes (including *Piezo1*), which are hypothesized to contribute to nocturia and enuresis (20, 39, 68, 69). Although our studies did not directly address the clock gene–regulated aspects of *Piezo1* expression, we did observe that loss of *Piezo1* expression alone results in nocturia in male mice and that disruption of voiding behavior in *Piezo1/2*-KO (and *Piezo2*-KO) mice is primarily altered during their active, dark phase. Our insights, combined with the tools and techniques we have developed, not only will enhance our understanding of the roles of urothelial mechanotransduction in LUT interoception but also should guide future investigations into the roles of PIEZO1/2 in normal and abnormal LUT function and the roles of sex and circadian rhythmicity in these events.

## Methods

### Reagents including antibodies.

Unless otherwise specified, all chemicals were reagent grade or better and obtained from MilliporeSigma. Primary antibodies were as follows: rat monoclonal anti-CDH1 (E-cadherin, clone DECMA-1; catalog number MABT26, MilliporeSigma); goat polyclonal anti–m-Cherry (catalog number MBS448057; MyBioSource); chicken polyclonal anti-KRT5 (catalog number 905901, BioLegend); rabbit polyclonal anti-KRT5 (catalog number 905501, BioLegend); rabbit polyclonal anti-KRT20 (catalog number ab53120, Abcam); rabbit polyclonal anti-RFP (catalog number 600-401-379; Rockland Immunochemicals); rabbit polyclonal anti-TJP1 (ZO-1; catalog number 61-7300, Thermo Fisher Scientific); goat polyclonal anti-TP63 (p63/TP73L; catalog number AF1916, R&D Systems, Bio-Techne); and rabbit polyclonal pan-uroplakin, a gift from T.-T. Sun (New York University, New York, New York, USA) (70). Minimal cross-reactivity goat and donkey secondary antibodies conjugated to Alexa Fluor 488, Alexa Fluor 548, Cy5, or HRP were obtained from Jackson ImmunoResearch Laboratories.

### Animals.

Both female and male mice were used in our studies and purpose bred from the strains described below. Mice were housed in standard caging, with automatic water dispenser and dry mouse chow ad libitum. All experimental mice, female and male, were virgins and group housed after weaning (up to 5 females/cage or up to 4 males/cage). Breeding mice were never used for experiments. Nonpregnant does were housed up to 5 per cage. Male breeders were always housed individually when not breeding. Animals were harem bred. The bottom of the cage was covered in standard bedding and included a plastic igloo or running wheel and a small square of paper for shredding. The animals were held under a 12-hour light/12-hour dark cycle. The weights and ages of animals used in our studies are presented in Supplemental Table 1. Euthanasia of mice was accomplished by inhalation of CO_2_ gas, followed by thoracotomy or cervical dislocation.

To define PIEZO1 expression and distribution, *Piezo1^tdT/+^* reporter mice (strain number 029214, The Jackson Laboratory) were mated to generate homozygous *Piezo1^tdT/tdT^* mice or WT *Piezo1^+/+^* mice (25). To assess *Piezo2* expression, a *Piezo2* mouse reporter line (*Piezo2^GFP-IRES-Cre^*; strain number 027719, The Jackson Laboratory) was used (30). It expresses GFP fused to PIEZO2 and the Cre recombinase under the direction of the *Piezo2* promoter. *Piezo2^GFP-IRES-Cre^* mice were mated with Ai9 mice (strain number 007909, The Jackson Laboratory), which express CAG promoter–driven *TdT* expression upon Cre-mediated removal of a stop codon upstream of the *TdT* sequence. To generate urothelium-specific conditional KO mice, we used constitutive *Upk2*-driven Cre expression (*Upk2*^Cre+/–^, strain number 029281, The Jackson Laboratory) (34). By mating *Piezo1^fl/fl^* mice (strain number 029213, The Jackson Laboratory) (71) with *Upk2^Cre+/–^* *Piezo1^fl/fl^* mice, we generated *Piezo1*-control (*Piezo1^fl/fl^* *Upk2^Cre–/–^*) and *Piezo1*-KO mice (*Piezo1^fl/fl^* *Upk2^Cre+/–^*). Similarly, by mating *Piezo2^fl/fl^* mice (strain number 027720, The Jackson Laboratory) (30) with *Upk2^Cre+/–^* *Piezo2^fl/fl^* mice, we generated *Piezo2*-control (*Piezo2^fl/fl^* *Upk2^Cre–/–^*) and *Piezo2*-KO mice (*Piezo2^fl/fl^* *Upk2^Cre+/–^*). To produce urothelium-specific conditional double KO mice, *Piezo1^fl/fl^* mice were crossed with *Piezo2^fl/fl^* mice to create *Piezo1^fl/fl^* *Piezo2^fl/fl^* mice. In turn, these were mated with *Upk2^Cre+/–^* *Piezo1^fl/fl^* *Piezo2^fl/fl^* mice to generate conditional urothelial *Piezo1/2*-control (*Piezo1^fl/fl^* *Piezo2^fl/fl^* *Upk2^Cre–/–^*) or *Piezo1/2*-KO mice (*Piezo1^fl/fl^* *Piezo2^fl/fl^* *Upk2^Cre+/–^*). In all experiments, non–Cre-expressing, sex-matched littermates were used as controls. Age-matched animals were used when littermates were not available. Genotyping was performed on tail snips collected from 21- to 25-day-old pups. Tail DNA was extracted using the QuickExtract DNA Extraction Solution (QE09050; Lucigen) and analyzed using the primer sequences and PCR protocols described by The Jackson Laboratory to confirm the animals’ genotypes.

### Detection and quantitation of Piezo channel expression using FISH.

To analyze gene expression of *Piezo1*, *Piezo2*, and *Upk3a* in the urothelium, we employed the RNAscope multiplex fluorescent kit (ACD) and the following probes: Mm-Piezo1 in channel 1 (catalog number 500511), Mm-Piezo2 in channel 3 (catalog number 400191), and Mm-Upk3a in channel 2 (catalog number 505891). Details about controls, the staining protocols, and quantitation are provided in the Supplemental Methods.

### Western blot analysis of tdT-PIEZO1 expression in bladder fractions.

Methods for Western blotting are described in the Supplemental Methods.

### Immunofluorescence labeling and tissue imaging.

Details of tissue recovery, fixation, freezing, sectioning, immunolabeling, and data acquisition are provided in the Supplemental Methods.

### RT-qPCR analysis.

Preparation of samples, primers, and details of the methods are provided in the Supplemental Methods. Relative gene expression was assessed using the 2^-ΔΔCt^ method (72).

### Peeled bladder and urothelial sheet preparations.

A peeled bladder preparation, similar to that described by Durnin et al., was made (73). A description of the methods to prepare peeled bladders and urothelial sheets is provided in the Supplemental Methods.

### [Ca^2+^]_i_ measurements, including those in response to cell poking.

An adenovirus encoding the Ca^2+^ sensor GCAMP5G was prepared, and mouse bladders were transduced using our established protocols (74, 75). As this technique involves transurethral catheterization, only female mice were used in these experiments. After 48 hours, the mice were euthanized, and a urothelial sheet preparation was made. The Krebs buffer was replaced with recording solution (135 mM NaCl, 5.0 mM KCl, 1 mM MgSO_4_, 2 mM CaCl_2_, 10 mM glucose, 10 mM HEPES, pH 7.4, gassed with 100% O_2_), and the culture dish containing the sheet preparation was mounted in a DH-35iL culture dish incubator (Warner Instruments). The tissue was continuously perfused with recording solution using an in-line heater (model SH-27B; Warner Instruments). The temperature of the chamber and solutions was maintained at approximately 37°C with a dual-channel bipolar temperature controller (model TC-344B; Warner Instruments). Images of GCAMP5G-transduced umbrella cells were acquired using a Hamamatsu ORCA fusion digital CMOS camera, coupled to an upright BX51W Olympus microscope, equipped with a Lambda XL light source (Sutter Instrument). To measure poking-induced changes in [Ca^2+^]_i_, individual umbrella cells expressing GCAMP5G were mechanically stimulated using a glass micropipette. The micropipettes were pulled from Corning 7056 glass (Warner Instruments), and the tip was fire polished and sealed to a diameter of approximately 1–3 μm using a Narishige MF-830 Micro Forge. The micropipettes were attached to a pipette holder, which was coupled to a Thorlabs piezoelectric actuator (model PAS005), which was itself mounted on an MP-285 micromanipulator (Sutter Instrument). To poke umbrella cells, the micropipette, positioned next to the cell’s apical surface, was programmed to move 20 μm, hold for a 1-second duration, and then return to its starting position. Poking was triggered by a signal from an open-loop piezo controller (model MDT694B, Thorlabs), remotely operated through a Digidata 1440A controller (Molecular Devices). Images were acquired every 185 ms and analyzed using cell-Sens software (Olympus) running on a Precision 5820 tower computer (Dell) outfitted with an Intel Xeon W-2145 3.7 GHz processor. The change in fluorescence intensity is denoted as ΔF/F, where F is the basal intensity of GCAMP5G at *t* = 0 and ΔF is the difference between the evoked fluorescence intensity and the basal intensity. In some cases, 30 μM Yoda-1 (catalog number 5580/10; Tocris, Bio-Techne), prepared as a 30 mM stock in DMSO, was added to the perfusate bathing the bladder sheet preparation, and images were acquired every 5 seconds.

### Measurement of serosal ATP release.

A peeled bladder preparation was made; residual fluid was removed from the peeled bladder lumen using a catheter attached to a 5 mL syringe. Subsequently, a 1 mL syringe, filled with Krebs-HEPES buffer (Krebs buffer supplemented with 10 mM HEPES, pH 7.4, and oxygenated by bubbling pure O_2_ gas through the solution), was attached to the catheter hub of the bladder preparation. The peeled bladder, attached to a micromanipulator, was submerged in 1 mL of Krebs-HEPES buffer that was contained within a custom, 3D-printed epoxy cylindrical chamber (inner diameter = 1.0 cm; height = 2.5 cm) with integral rectangular base (1.8 × 4.0 cm). The base was designed to fit in the slot of a PM-5 heated platform with magnetic clip (Warner Instruments). The PM-5 was heated by a TC344B dual automatic temperature controller (Warner Instruments). The chamber was perfused through an inlet with Krebs-HEPES buffer at a flow rate of 1.5 mL/min using a Harvard Apparatus PHD-Ultra syringe pump. The buffer entering the chamber was heated to 37°C using a model SH-27B (Warner Instruments) in-line heater connected to the TC-344B unit. The cylindrical chamber also had a side port, which was connected to a Gilson Minipulse 3 pump (flow rate = 1.5 mL/min), which released fluid from the chamber as the volume approached approximately 1 mL. The effluent from the pump was collected into 5 mL sterile screw-cap tubes (free of DNAse, RNAse, apyrase, and Cytoxan; catalog number C2540, MTC Bio). During experiments, the peeled bladders, mounted in the setup, were flushed with Krebs-HEPES buffer for 20 minutes, then for 10 minutes with Krebs-HEPES buffer supplemented with 30 μM Ebselen. Three 1.5 mL aliquots were collected in the 5 mL tubes, and then the bladder was filled over a 10-second period with Krebs-HEPES-Ebselen buffer. The volume used to fill the bladders varied with the age of the animals, as bladder size varies: 120 μL in female or 150 μL in males that were 7–11 weeks old. However, this amount was increased to 200 μL in all animals more than 12 weeks old. Subsequently, a sample was collected every minute (~1.5 mL), for 12 minutes. Immediately after each sample was collected, it was heated at 98°C for 2 minutes and then frozen in liquid nitrogen. The samples were recovered from the liquid nitrogen and stored at –20°C until ATP measurements were made. Details of the ATP assays can be found in the Supplemental Methods.

### Muscle strip analysis.

These analyses were performed as described previously (76), using the modifications described in the Supplemental Methods.

### Cystometry.

Mouse voiding function was assessed by cystometry using our previously described methods (77), with modifications described in the Supplemental Methods.

### Video-monitored void spot assay.

To evaluate voiding behavior in awake, freely moving mice, we modified the standard void spot assay to incorporate video monitoring, akin to the setup described by Keller et al. (78) (Figure 4A). By incorporating real-time video monitoring into these assays, we could follow mouse activity over extended periods of time while overcoming the difficulties of distinguishing overlapping voiding spots (a result of mice urinating multiple times in the same location), an oft-cited shortfall of these assays (40). Moreover, we could readily detect small voiding events, confirming that they were not a result of nonspecific carryover from the mouse’s fur or trailing associated with larger void spots. The system consisted of a custom-made frame with 2 compartments, constructed of T-slot aluminum framing material, connectors, and panels (80/20 Inc.). The upper compartment (dimensions of 37 × 25 × 20 cm) was divided in half to house 2 mice in a side-by-side configuration. The top, side walls, and bottom of the upper chamber were made of clear UV radiation–transmitting acrylic plastic. The side wall between adjacent animals was covered with opaque paper, and the bottom of the upper compartment was covered in its entirety with blotting paper (see below), which was illuminated from below by two 24-inch-long UV tube lights (model T8-F20BLB24; ADJ Products LLC) mounted to the floor of the lower compartment, but with light directed upward. Reflecting differences in our ability to visualize spots, we used 2 different papers: cosmos blotting paper (catalog number 10422-1005; Blick Art Materials) was used to monitor voiding behavior during the dark phase, whereas chromatography paper (catalog number 057144, Thermo Fisher Scientific) was used during the light phase. The lower compartment of the chamber, made of mirrored plexiglass panels (80/20 Inc.), allowed the UV light to reflect light upward. Mouse activity and voiding behavior were monitored using wide-angle webcams (model C930e, Logitech): one positioned above the cage and another mounted at the base of the lower compartment. The upper camera was most useful to monitor events in the dark phase, while the lower camera was most useful during the light phase. Each mouse compartment was furnished with the following: an igloo-shaped sleeping chamber, an Eppendorf tube as enrichment, and a dish with standard mouse chow and water in the form of Hydrogel (ClearH2O). The mice were routinely housed in a facility with 12-hour light/12-hour dark cycle, with 7 am being zeitgeber time (ZT) = 0 (start of light cycle). To analyze their voiding behavior in the dark phase, the mice were placed in the upper chamber between ZT = 10 and 11, and analysis of void spots was performed from ZT = 17 to ZT = 2 3. The extended period of acclimatization reflected access limitations to the facility after 7 pm. For analysis during the light phase, the mice were placed in the chamber between ZT = 3 and 4, then allowed to acclimatize for 1 hour, and analysis was performed during the subsequent 6-hour time window (ZT = 4–5 to ZT = 10–11). Video was captured at 1 frame per second with a 1920 × 1080 pixel resolution using an Apple iMac computer running SecuritySpy software (Ben Software). The movies were saved in.M4V format and viewed on an Apple iMac computer using Quicktime (Apple) software.

Voiding events were identified by visual inspection of the movies. Males had a tendency to “walk” while voiding, giving the impression of multiple spots/smear for a single voiding event. If confirmed to be a single voiding event by video, it was recorded as such. To demonstrate that voiding behavior was voluntary in nature, we confirmed that the mice moved to the area of interest, urinated, and then left. When a voiding event was observed, the video was played frame by frame to capture the exact time of voiding and the frame in which the urine spot had diffused maximally. A screenshot of the entire video frame was made and the resulting TIF file opened in NIH ImageJ. The Freehand tool was used to select the boundary of the spot. The same tool was used to select the perimeter of the paper. The ratio of spot area to paper area was recorded. Calibration curves, made by spotting mouse urine (2–750 μL) on the appropriate paper type, were similarly analyzed and used to calculate the volume/void spot. Consistent with previous reports, spots were categorized as PVSs (those ≥20 μL) or SVSs (<20 μL) (43). The parameters measured in our analysis were number of PVSs, average volume per void, total PVS volume, number of SVSs, and total SVS volume. Urinary continence scores were calculated as described by:


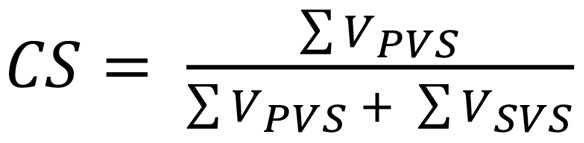


where 
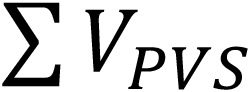
 is the sum of all PVS volumes and 
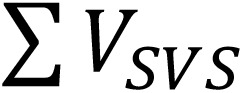
 is the sum of all SVS volumes. The following animals were excluded from analysis: aggressive, dominant alpha male mice; those with more than 100 voids during the dark phase or more than 50 voids during the light phase; and those animals that chewed or damaged the paper prior to or during the time window of analysis.

### Statistics.

Data are reported as mean ± SEM (*n*), where *n* equals the number of independent experiments or individual animals. In experiments where multiple, replicate values were measured (e.g., during cystometry), the values for an individual mouse were averaged prior to assessing statistics for the sample population. Parametric or nonparametric tests were employed based on the results of the Shapiro-Wilk and Kolmogorov-Smimov normality tests. If data were normally distributed, then 2-tailed *t* tests were performed, using Welch’s correction if the variance between groups was unequal. If data were not normally distributed, then a Mann-Whitney *U* test was employed. *P* ≤ 0.05 was considered statistically significant. Statistical analyses were performed using GraphPad Prism 8/9 (GraphPad Software).

### Study approval.

All animal studies were performed in accordance with relevant guidelines/regulations of the Public Health Service Policy on Humane Care and Use of Laboratory Animals and the Animal Welfare Act and under the approval of the University of Pittsburgh Institutional Animal Care and Use Committee.

## Author contributions

MGD, GA, and MDC conceived the idea of studying the role of PIEZO channels in urothelial mechanotransduction. WGR established the peeled bladder preparation in our labs and developed the bladder sheet preparation. MDC designed the ATP assay and created the assay chamber to measure ATP release. MGD, NM, and WGR carried out experiments using the ATP assay chamber. MDC developed and performed experiments measuring poking-induced changes in intracellular calcium. MDC and NM conceived and oversaw the construction of the void spot chambers. MGD and WGR designed and carried out experiments using the void spot chambers. DRC, WGR, MGD, and GA conceived and executed experiments requiring image analysis. DRC performed image capture, image processing, and quantitation. WGR performed the electron microscopy analysis. JMB and SLD designed and helped execute along with MGD the muscle strip analysis. GA and MGD wrote the initial drafts of the manuscript, with subsequent contributions from all the other authors.

## Supplementary Material

Supplemental data

Supplemental video 1

## Version 1 08/31/2021

In-Press Preview

## Version 2 10/08/2021

Electronic publication
