# Supplementary material for: Functional roles for PIEZO1 and PIEZO2 in urothelial mechanotransduction and lower urinary tract interoception
Source: JCI Insight. 2021 Oct 8;6(19):e152984. doi: 10.1172/jci.insight.152984 (PMC8525643; doi:10.1172/jci.insight.152984)

Dalghi et al. SUPPLEMENTARY INFORMATION

Table S1: Mouse ages and weights*

**Genotype**	**Sex**	**Age (months)**	**Weight (g)** **Mean ± S.D.**	**n**
Piezo1-control	Female	2 - 6	22.9 ± 3.5	16
	Male	2 - 5	27.0 ± 3.3	16
Piezo1 - KO	Female	2 - 6	21.9 ± 2.6	16
	Male	2 - 5	27.2 ± 2.7	16
Piezo2-control	Female	1.5 - 3	19.2 ± 1.3	11
	Male	2 - 4	28.0 ± 1.8	9
Piezo2 - KO	Female	1.5 - 3	19.8 ± 1.6	13
	Male	2 - 4	26.8 ± 1.9	9
Piezo1/2-control	Female	1.5 - 3	18.6 ± 1.3	14
	Male	2 - 4	26.8 ± 2.2	12
Piezo1/2 - KO	Female	1.5 - 3	18.8 ± 1.5	14
	Male	2 - 4	25.8 ± 1.8	12

*These are the ages and weights for animals prior to analysis in void-spot assays. These animals were subsequently used in the other assays and protocols described in the manuscript.

SUPPLEMENTARY VIDEO LEGEND

Video 1. Poking-induced increase in $[Ca^{2+}]_i$ in urothelial cells expressing the Ca^{2+} indicator GCAMP5G. The cell indicated with a red arrow is poked in the video at the ~1.0s mark, stimulating an almost instantaneous increase in $[Ca^{2+}]_i$, which returns to baseline after ~13s. Adjacent umbrella cells, including those that are 60-100 µm away, also respond, but after a several second delay.

SUPPLEMENTARY METHODS

Detection and quantitation of *Piezo* channel expression using fluorescent *in situ* hybridization. To analyze gene expression of *Piezo1*, *Piezo2*, and *Upk3a* in the urothelium, we employed the RNAscope™ multiplex fluorescent kit (ACD; Newark, CA) and the following probes: Mm-Piezo1 in channel 1 (catalog number 500511), Mm-Piezo2 in channel 3 (catolog number 400191), and Mm-Upk3a in channel 2 (catalog number 505891). Probe diluent was used when channel 1 probe was not included in the staining protocol. The 3-plex negative control probe against *Bacillus subtilis dapB* (catalog number 320871) was used to detect non-specific signal. Bladders were prepared using a fresh-frozen approach. Procedures were performed using gloves and sterilized equipment to prevent RNAse contamination. In brief, mice were euthanized by CO_2 inhalation, their bladders excised, rinsed with 37˚ C Krebs buffer (110 mM NaCl, 25 mM $NaHCO_3$, 5.8 mM KCl, 1.2 mM $MgSO_4$, 1.2 mM KH_2PO_4, 11 mM glucose, 2 mM $CaCl_2$, pH 7.4 when gassed with 5% v/v CO_2), and then placed in a 10-cm square dish filled with the same buffer. A small amount (~50 µl) of Optimal Cutting Temperature embedding medium (OCT; Tissue-Tek, Sakura Finetek, Torrance, CA) was injected into the bladder lumen by way of the catheter/hub assembly of a 24G x ¾" Jelco Safety IV catheter (Smiths Medical ASD, Southington, CT) attached to a 1-ml syringe. The bladder was dipped into a beaker containing OCT, and then transferred to a 10mm x 10mm x 5mm Tissue-Tek Cryomold (Sakura Finetek USA Inc, Torrance, CA) filled with OCT. The bladder was oriented in the block so that the neck was facing downward. The tissue

was frozen by placing the blocks in a -80˚ C freezer and then stored at -80˚ C in sealed plastic bags. Sections (8 µm) were cut using a Leica CM1950 cryostat (chamber temperature of -20˚ C and a knife temperature of -18˚ C), collected on Superfrost™ Plus microscope slides (ThermoFisher Scientific, Waltham, MA), and "dried" by storing them in the cryostat chamber for 30 min, prior to long-term storage at -80˚ C. The ACD protocol was followed as described in their technical bulletin, but with the following changes. Slides containing sectioned tissue were removed from the -80˚ freezer, and immediately fixed for 15 min at 4˚ C with neutral buffered formalin comprised of 29 mM NaH_2PO_4•H2O, 45.8 mM Na_2HPO_4, and 4.0% (v/v) paraformaldehyde (neutralized to pH 7.2 while preparing a 40% w/v stock). For tissue processed for *Piezo1* probes, we employed Protease IV treatment for 5 min at room temperature, and for *Piezo2* probes we used Protease III treatment for 25 min at room temperature. The *Upk3a* probe worked in either case. We observed the least background signal, and most specific staining when the *Piezo* probes were developed in channel 2 using the TSA Plus CY3 reagent (diluted 1:750; catalog number FP1170012UG Perkin Elmer). The *Upk3a* probe was developed in channel 1 using the TSA Plus Cyanine-fluorescein reagent (diluted 1:1000; catalog number FP1168015UG, Perkin Elmer). Slides incubated with negative control probes were run side-by-side with those reacted with *Piezo1/2* probes and treated identically. Labeled tissue was mounted using ProLong Gold antifade mountant (ThermoFisher), cured overnight at room temperature in the dark, and slides subsequently stored at 4˚ C.

Fluorescent and corresponding DIC images were acquired using an HC PLAN APO 10X objective (N.A. 0.40) or an HCX PLAN APO 40X oil objective (N.A. 1.25) attached to a DM6000B widefield microscope (Leica Microsystems, Buffalo Grove, IL) outfitted with a Gryphax Prokyon digital camera (Jenoptik, Jupiter, FL). The multi-fluorescence capture mode of Gryphax software (Jenoptik) was used to collect images (1920 x 1200 pixels). For quantitation, random cross sections through the bladder were photographed in their entirety using the 10X objective. Photomontages were created using Photoshop CC2021 (Adobe, Mountain View, CA) to "stitch"

the images using the File > Automate > Photomerge function. Upon opening the images at 72 dpi, a grid was placed over the merged images using the Photoshop > Preferences > Guides, Grids & Slices option to set the gridline every 5.52 cm. A random number table was used to identify 5 regions of the photomerged image that contained urothelium, which were then re-photographed under the microscope using the 40X objective to generate high-magnification images (cropped to 1200 x 1200 pixels to reflect the square nature of the gridlines). To quantify *Piezo1* expression, the high-magnification images were opened in Fiji. Using the DIC image and *Upk3a* signal as references, the boundary of the urothelium was outlined using the Freehand Tool, and the area calculated using a calibrated length standard and the Analyze > Set Scale function. The number of *Piezo1* fluorescent punctae were counted and reported as dots per area of sampled urothelium. In the case of *Piezo2*, we used a different counting strategy, as only a small number of umbrella cells (and few if any intermediate/basal cells) were positive for *Piezo2* expression. After identifying and photographing 5 regions in the photomerged image, we measured the length of luminal surface using the Freehand Line tool of Fiji (calibrated as described above). We then counted the *Piezo2*-positive umbrella cells, reporting the number of positive umbrella cells per length of luminal surface. Figures were prepared from representative images using the techniques described below.

Western Blot analysis of tdT-PIEZO1 expression in bladder fractions. *Piezo1*$^{tdT/tdT}$ or *Piezo1*$^{+/+}$ mice were used in these studies. To remove PIEZO1-rich red blood cells, the euthanized animal's vasculature was perfused with 10 mL of 37˚ C phosphate buffered saline (PBS; 137 mM NaCl, 2.7 mM KCl, 8 mM Na_2HPO_4, 1.5 mM KH_2PO_4, pH 7.4) injected via syringe attached to a 26G needle inserted into the heart's left ventricle, with blood allowed to escape from the transected right pulmonary artery. Subsequently, a "peeled bladder" preparation was made (see methods below) to isolate two bladder fractions: mucosal (comprised of urothelium and subjacent connective tissue) and detrusor (remaining connective tissue and smooth muscle layers). The

tissue in each fraction was minced with scalpels, lysed, resolved by PAGE, and PIEZO1-tdT detected by Western blotting and quantified using our previously described methods (22).

Immunofluorescence analysis. The kidneys, ureters, bladder, and urethra were recovered from euthanized animals. Kidneys were bisected along their medial, transverse/axial plane using a fresh razor blade. The tissues were fixed by immersion in 4% w/v paraformaldehyde (dissolved in 100 mM phosphate buffer, pH 7.4). After 5 min, fixative (~100 µl) was gently flushed into the urethra and lumen of the bladder by use of a yellow-tip attached to a P200 Gilson (Madison, WI) pipettor. The tissue was fixed for a total of 60 min. In the case of whole-mounted tissue, the fixed bladders were rinsed with PBS and then immunolabeled. For cryosections, the fixed tissue was cryoprotected in 35% (w/v) sucrose dissolved in PBS at 4˚ C until the tissue sank. The sucrose-impregnated tissues were placed in OCT-filled cryomolds, oriented in the following manner: kidney pieces, cut surface down; bladder, neck region facing down; ureters, long axis perpendicular to the bottom of the mold; urethra, proximal region facing downwards. Immunolabeling was performed by incubating cryosections, or intact tissue, for 10 min at room temperature in Quench Buffer (75 mM NH_4Cl and 20 mM glycine, pH 8.0 dissolved in PBS, containing 0.1% v/v Triton X-100), rinsing three times with PBS, and then three times for 5 min with PBS, followed by incubation in Block Solution (PBS containing 0.6% v/v fish skin gelatin, 0.05% w/v saponin) containing 5% v/v donkey or goat serum for 60 min at room temperature. The samples were subsequently incubated with primary antibodies (diluted in Block Solution) for 2 h at room temperature or overnight at 4 °C in a humid chamber. The slides were washed 3 times quickly and 3 times for 5 min with Block Solution, and then incubated with minimal cross-reactivity, fluorophore-labeled secondary antibodies, diluted in Block Solution, for 1 h at room temperature. In some cases, nuclei were counterstained with TO-PRO-3 (1:1000; ThermoFisher Scientific) and overall tissue architecture visualized using tetramethylrhodamine isothiocyanate (TRITC)–labeled phalloidin (ThermoFisher Scientific; 1:200). The labeled tissues were then rinsed 3 times quickly

and 3 times for 5 min with Block Solution, rinsed with PBS, and then postfixed in 4% paraformaldehyde/phosphate buffer for 5-10 min at RT. The slides were rinsed with PBS, and the samples mounted under borosilicate coverslips (#1.5, 0.17 mm thickness, 24 x 50 mm; ThermoFisher) using SlowFade Diamond Antifade mounting medium (ThermoFisher). The edges of the coverslip were sealed with clear nail polish, and the slides stored at -20 °C until image acquisition was performed.

Images were captured using a Leica HCX PL APO 20X or a Leica HCX PL APO CS 40X, 1.25 NA oil objective and the appropriate laser lines of a Leica TCS SP5 CW-STED confocal microscope (in normal confocal mode). The photomultipliers or HyD detectors were optimized using the Q-LUT option, and 8-bit images collected using 8 line averages combined with 4 frame averages. Crosstalk between channels was prevented by use of spectral detection coupled with sequential scanning. Stacks of images (1024 x 1024, 8-bit, typically 3-6 images) were collected using a Z-step of 0.29 μm. Images were imported into Volocity 4-D software (Perkin Elmers; Waltham, MA), and following image reconstruction, exported as TIFF files. The contrast of the images was corrected in Photoshop CC2021 (Adobe; San Jose, CA), and composite images prepared in Adobe Illustrator CC2021. Representative images are shown in each figure.

RT-qPCR analysis. Excised bladders were placed in a dish filled with Krebs solution and the region of the bladder neck closest to the urethra removed using a scalpel. The orifice of a 10 μl Pipettman white tip (Gilson) was widened by removing 2 mm of plastic from its tip (using a scalpel). It was then positioned so that it touched the apex of the bladder dome region. Using forceps to grab the bladder's cut end, the bladder was inverted onto the white tip, exposing the urothelium. The "inverted bladder" was immersed in 150 μl of lysis/binding buffer (RNAqueous-4PCR Kit; Invitrogen, Waltham, MA) for 30 s, and total RNA was purified using the manufacturer's protocol. cDNA was generated using random primers provided in the AccuScript pfuUltra II RT-

PCR kit (Agilent, Santa Clara, CA). The synthesis step was 45 min. The relative expression of mRNA was quantified by qPCR in a GFX Connect real-time PCR (Bio-Rad, Hercules, CA) using TaqMan (Applied Biosystems, ThermoFisher Scientific) reagents for Piezo1 (probe number Mm01241549_m1) or β-actin (probe number Mm00607939_s1) and the TaqMan Fast Advanced Master Mix (catalog number 4444553; Applied Biosystems, ThermoFisher Scientific). Cycling conditions were 50°C for 2 min and then 95°C for 10 min, followed by 40 cycles of 95°C for 15 s and 60°C for 1 min. Samples were run in triplicate. Relative gene expression was analyzed using the 2-ddCt method (72). The qPCR machine was operated by and Ct values determined using BioRad CFX Manager 3.1 software (Hercules, CA).

Peeled bladder and urothelial sheet preparations. Euthanized mice were placed in a supine position, and a midline incision made from the pelvis to 1 cm below the sternum to expose the pelvic bone, bladder, and abdominal cavity. The pelvic bone was cut along its midline using scissors, and pulled away from the cut site using tweezers, exposing the urethra. The plastic catheter/hub assembly of a 24G x ¾" Jelco Safety IV catheter (Smiths Medical ASD) was inserted into the urethra until its tip was positioned just below the bladder neck. Prior to insertion, and to prevent introducing air into the bladder, the catheter was filled with warm Krebs buffer via a syringe attached to the catheter hub. The catheter was held in place by looping 6-0 silk suture material (Covidien, Minneapolis, MN) around the tissue surrounding the catheter below the base of the bladder, and securing it using overhand knots. Two additional loops of suture material were placed along the urethra, positioning the final one close to the external meatus. Sharp scissors were used to liberate the bladder, urethra, and associated catheter from the underlying tissues. The preparation was placed in a dish filled with Krebs buffer, which was kept at 37˚ C by way of a heating plate. The submerged bladder was partially filled by injecting 75 µl of Krebs buffer from an attached syringe into its lumen, then the detrusor and surrounding connective tissue were carefully dissected from the urothelium, starting at the neck region, using scissors and tweezers,

flipping the preparation midway to work on the reverse surface of the bladder. The peeled bladder served as a prequel to generating urothelial sheets. In this case, the peeled bladder was severed from the catheter hub using sharp scissors, and an incision made along the neck-to-dome axis. The cut-open, peeled bladder was carefully pinned out using 0.15-mm stainless steel Minutien insect pins (Carolina Biological Supply Company, Burlington, NC) to a 5-mm layer of Sylgard™ 184 silicone elastomer (Dow Silicones Corporation, Midland, MI) polymerized on the bottom of a 35-mm tissue culture dish. The remainder of the dish was filled with 37˚ C Krebs buffer, in which the bladder tissue was submerged throughout the process.

ATP measurements: To measure ATP in the samples, a luciferin-luciferase assay employed. Briefly, 10 µl of the sample was placed in a Nunc F96-microwell white polysytrene dish (catalog number 236105, ThermoScientific), and 90 µl of reaction mixture added. Reaction mixture was comprised of 0.5 mM D-luciferin (catalog number L2912, Invitrogen) and 4 µg/ml Quantilum recombinant luciferase (catalog number E1701, Promega, Madison, WI) diluted in reaction buffer (25 mM Tricine, pH 7.8, 5.0 mM $MgSO_4$, 0.1 mM EDTA, and 0.01% w/v NaN_3). Samples were quantified using a Glomax Multi+ detection system multi-plate reader (Promega) with a 5-s integration period and gathered using Instinct Software. The raw data were given in RLU (relative light units) and converted to pmol ATP using a standard curve (0-16 nM ATP), which was generated and run side-by-side each experiment.

Muscle strip analysis. Mice were euthanized, the bladder excised, and then placed on a silicon mat in a 15-cm round dish filled with warm Krebs buffer under continuous bubbling with 95% v/v O_2 / 5% v/v CO_2. The bladder was oriented horizontally with its neck portion in proximity to the experimenter and pinned at the dome to the surface of the mat. The bladder was cut open longitudinally by inserting the scissors through the neck orifice and then pinned at its four vertices with the luminal side facing upwards. Using a scalpel, each bladder was cut longitudinally into two

equal strips. Each bladder strip was mounted on a glass rod on its distal neck end and hooked to a tension transducer from its dome region using silk suture material. The strips were placed in a 20 mL water-jacketed organ bath filled with 15 mL Krebs buffer maintained at 37˚ C under agitation and with continuous bubbling with 95% v/v O_2 / 5% v/v CO_2. The strips were adjusted to a tension of 0.5 gram (5 mN) and allowed to equilibrate for 1 hour before treatments were applied. The force-displacement transducer (model FT-03, Grass Instruments/Astro-Med Inc., West Warwick, RI) was calibrated using no weight as a baseline or in response to a 5-gram calibration weight. After equilibration, the bladder strips, placed between two platinum electrodes, were subjected to electric field stimulation (EFS) using a Grass stimulator (model S88; Grass Instruments/Astro-Med Inc.). Successive pulses, with frequencies from 1Hz-32Hz at 70V potential, were applied in train (1.5 ms duration and 1.5 ms delay) for 5s, followed by a 30s rest before increasing the frequency of the pulse. After EFS stimulation, strips were incubated with increasing carbachol concentrations (1 nM to 10 μM final concentrations) that were added to the solution bathing the strips with 5 min intervals and responses recorded. Data was acquired using the WinDaq software (DATAQ Instruments Inc., Akron, OH) running on a Windows 10-based computer (Dell Technologies, Round Rock, TX). After finishing the experiment, the strips were removed from their associated rod, excess liquid removed gently with tissue paper, and the wet tissue weight recorded.

Data were analyzed as follows. The response of the bladder strips to EFS and carbachol was calculated as the amplitude of the contraction. In the case of EFS, the amplitude of the contraction was calculated as the difference between basal value (the minimum value of 6s before first stimulus is applied) and the peak after each impulse (the maximum value within the 6s after a stimulus is applied). For carbachol experiments, the baseline is the minimum value within the 5 min before the first addition, and the maximum contraction is the maximum value within the 5 min after addition. In both cases, for EFS and carbachol stimuli, raw amplitude values were corrected

by the weight of each strip and one average value was obtained for the two strips. Values were plotted as % of the maximum contraction obtained for the control, which is considered 100%.

Cystometry. Mice were anesthetized with isoflurane (1.5%) and then urethane (prepared as a 180 mg/ml stock in water and filter-sterilized) was injected subcutaneously in the abdominal region at 1.2g/kg body weight. The isoflurane was halted, and the animals allowed to rest in the dark surrounded by warm water packs until they were fully anesthetized and no longer responsive to a toe pinch. The mice were placed in a supine position, a midline laparotomy was performed, and the bladder exteriorized. The bladder was held in place by hooking the end of a bent needle, suspended above the mouse, to a small portion of the serosal tissue encapsulating the dome region of the bladder. The bladder was moistened with normal saline and shallow purse string sutures were made around the dome using Unify 6–0 silk suture material (catalog number S-S618R13, AD Surgical, Sunnyvale, CA), and the area within the boundary of the sutures was punctured using a sterile 19G needle. PE-50 tube was flame-flanged, and the flared end inserted into the hole created by the 19G needle. The suture was tightened around the tubing and the tube was gently retracted until < 1 mm of tubing was left in the bladder lumen and the flange was flush with the mucosa. The bladder was returned to the peritoneal cavity, and the surgical incision was closed around the free end of the PE-50 tubing using 6–0 silk suture to close the tissue layers and skin. The exteriorized end of the PE-50 tubing was connected to Y connector, with one branch attached to a pressure transducer (MLT844; ADInstruments, Colorado Springs, CO) and the other branch connected to a syringe filled with Krebs-HEPES buffer. The pressure transducer was linked to a Quad Bridge Amplifier, which was interfaced to a Powerlab 4/30 A/D converter (ADInstruments) that output data to an connected iMac computer (Apple,Cupertino, CA) running the Chart 5.0 program (ADInstruments). The pressure responses were calibrated by lowering and raising a 50-cm column of water attached to each pressure transducer prior to the start of the experiment. The syringe attached to the bladder catheter was placed in a Harvard Apparatus

PHD Ultra syringe pump (Holliston, MA) and set to deliver fluid at a rate of 15 µl/min. After a one-hour period of equilibration, data were collected for at least another hour (~8-10 successive cycles of filling/voiding). In female mice, the effluent was collected at each void, and the total recovered volume recorded. In male mice, voiding contractions were recorded, but as it is difficult to accurately recover the expelled buffer from males, we could not calculate voiding efficiency.

The following parameters were measured (refer to Fig. 3 for sample cystometrogram): 1) the resting pressure (RP) was defined as the mean pressure recorded 10 s after a void; 2) the threshold pressure (TP) was defined as the mean pressure that was recorded 5-10s before the rapid rise if pressure that accompanies a voiding event; 3) the peak pressure (PP) was the maximal contraction pressure recorded during the voiding event; (4) the amplitude of contraction (A) was the difference in peak pressure and threshold pressure (i.e., A = PP - TP); 5) bladder compliance (∆volume/∆pressure) was computed using the following formula:

$$C = \frac{Q \times t_{RP-TP}}{\Delta P_{RP-TP}}$$

where C is equal to compliance, Q is the flow rate, t_{RP-TP} is the time interval between RP and TP, and ΔP_{RP-TP} is the difference in pressure between RP and TP; 6) the intervoid interval (IVI) was the time between RP and TP. Values for successive 8-10 filling/voiding cycles were averaged, and mean values reported for each animal. (7) The voiding efficiency was determined by dividing the total recovered volume vs. the nominal amount of buffer infused into the bladder.

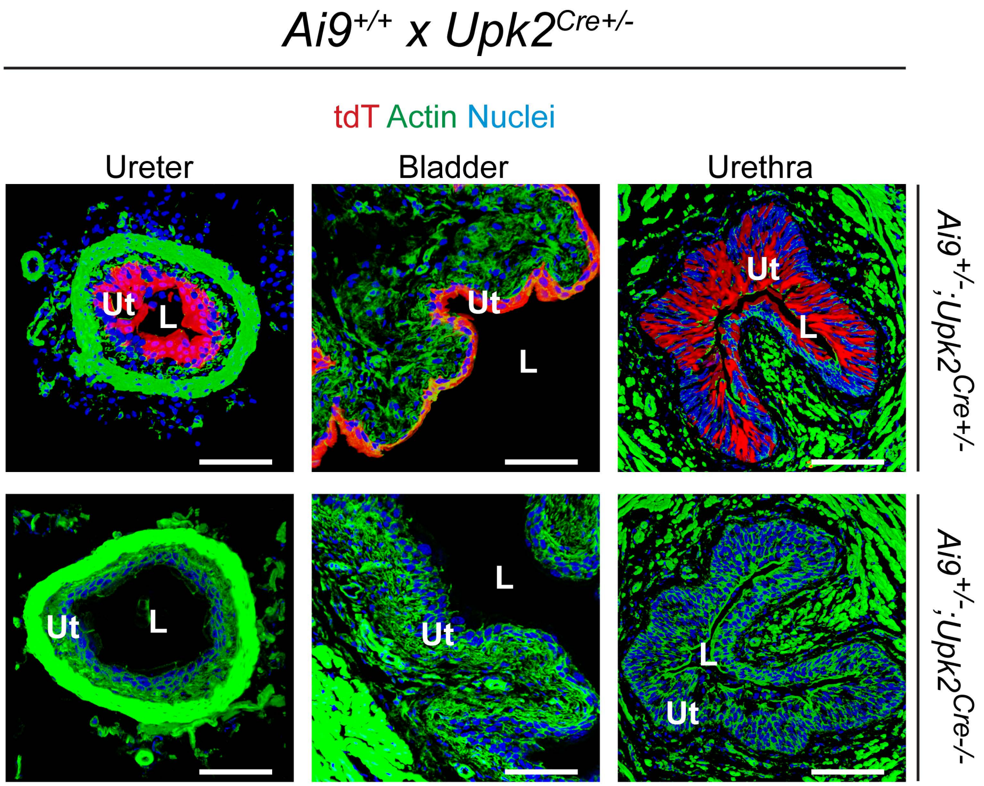

Figure S1. *Upk2*-Cre-mediated expression of tandem-dimer tomato (tdT) in the urothelium. Ai9 mice were bred with *Upk2*$^{Cre+/–}$ mice and expression of tdT (red) was detected using immunofluorescence and confocal microscopy. Tissue sections were counterstained with FITC-phalloidin to label the actin cytoskeleton (green) and To-Pro-3 to label nuclei (blue). Images are 3D reconstructions of 29-72 optical sections. Key: L, lumen; Ut, urothelium. Scale bars are 100 μm.

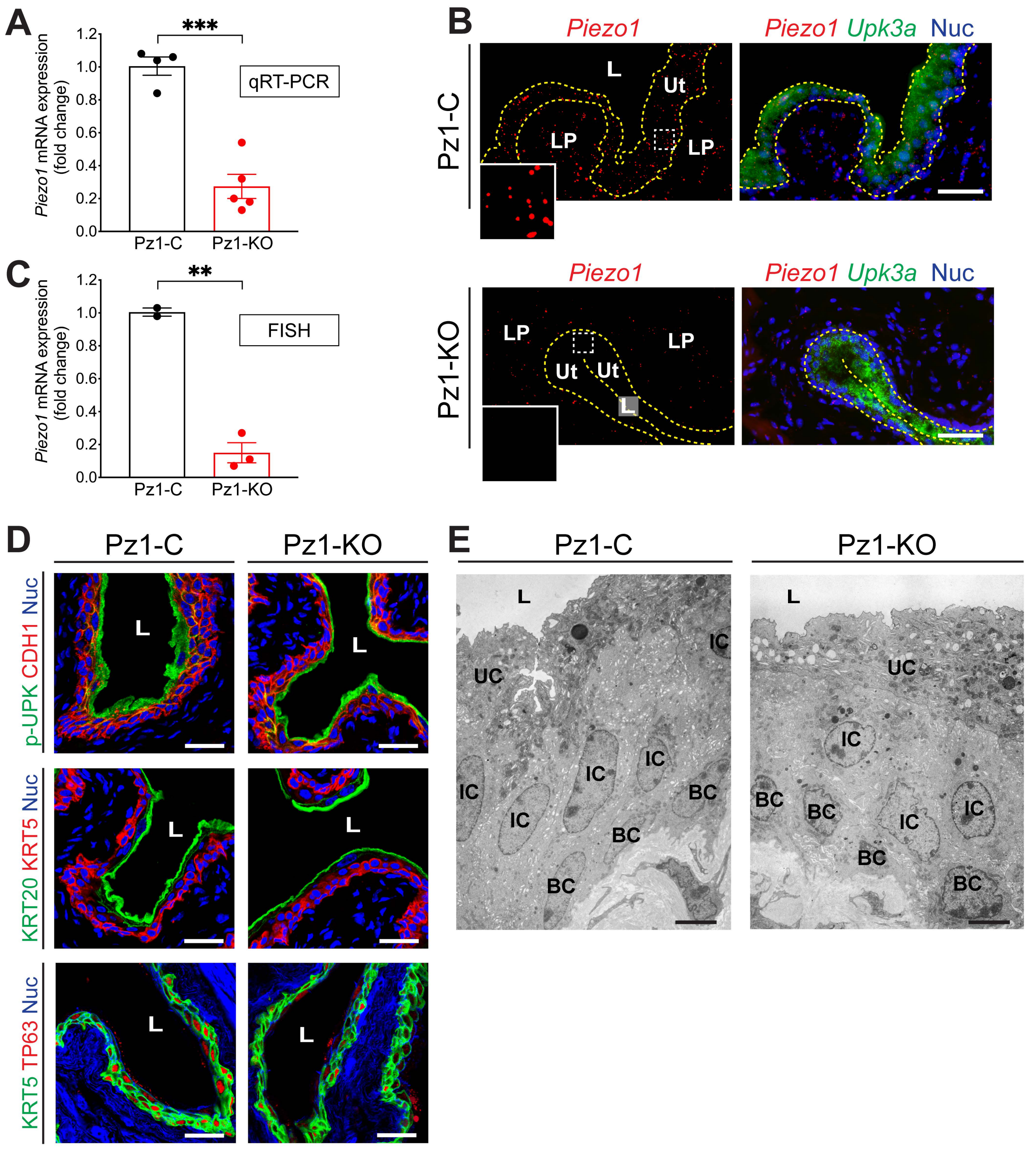

Figure S2. Characterization of *Piezo1*-KO mice. **(A)** Expression of *Piezo1* mRNA in *Piezo1*-control and *Piezo1*-KO mouse urothelium assessed by qRT-PCR. Data were analyzed using t-tests, and significant differences marked with a triple asterisk ($p \leq 0.001$). **(B)** FISH analysis of *Piezo1* and *Upk3a* expression in the indicated mouse strains. The boxed area surrounded by a white dashed line is magnified in the inset. The boundaries of the urothelium are indicated by yellow dashed lines. Key: L, lumen; LP, lamina propria; Ut, urothelium. Scale bars are 50 μm. **(C)** The number of *Piezo1* dots per urothelial area were quantified using FISH and normalized to data obtained for *Piezo1*-control animals. Data were analyzed using t-tests, and significant differences indicated by a double asterisk ($p \leq 0.01$). **(D)** Expression and distribution of uroplakins (detected using a pan-uroplakin antibody), CDH1, KRT5, KRT20, and TP63 in Pz1-C and Pz1-KO urothelium assessed using immunofluorescence. Images, obtained using a confocal microscope, are 3D reconstructions of 7-17 optical sections. L = lumen. Scale bars are 40 μm. **(E)** Ultrastructure of Pz1-C and Pz1-KO urothelium assessed using transmission electron microscopy. Key: BC, basal cell; IC, intermediate cell; L, lumen; UC, umbrella cell. Scale bars are 5 μm.

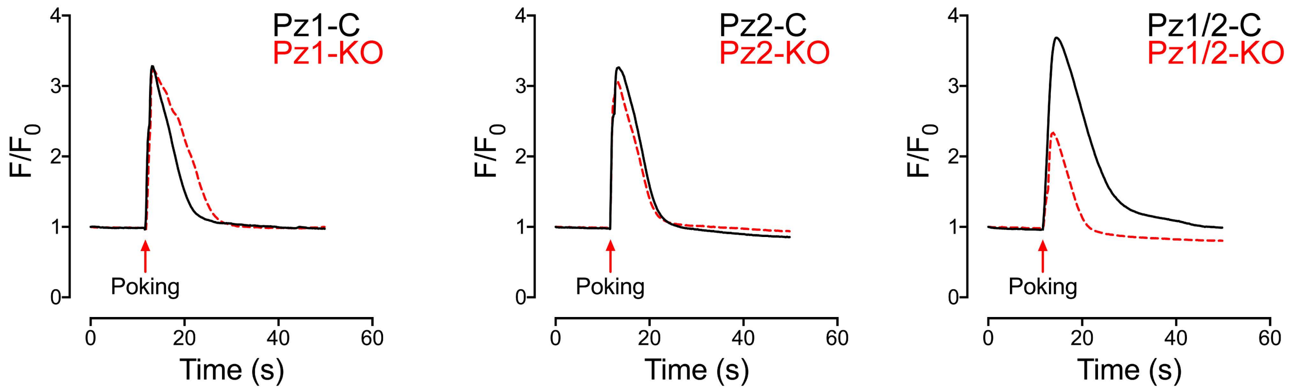

Figure S3. Poking-induced increases in $[Ca^{2+}]_i$ in the urothelium of the indicated mouse strains transduced with adenovirus encoding GCAMP5G. Data are representative traces from a total of three mice per strain, with 11-14 umbrella cells poked per mouse.

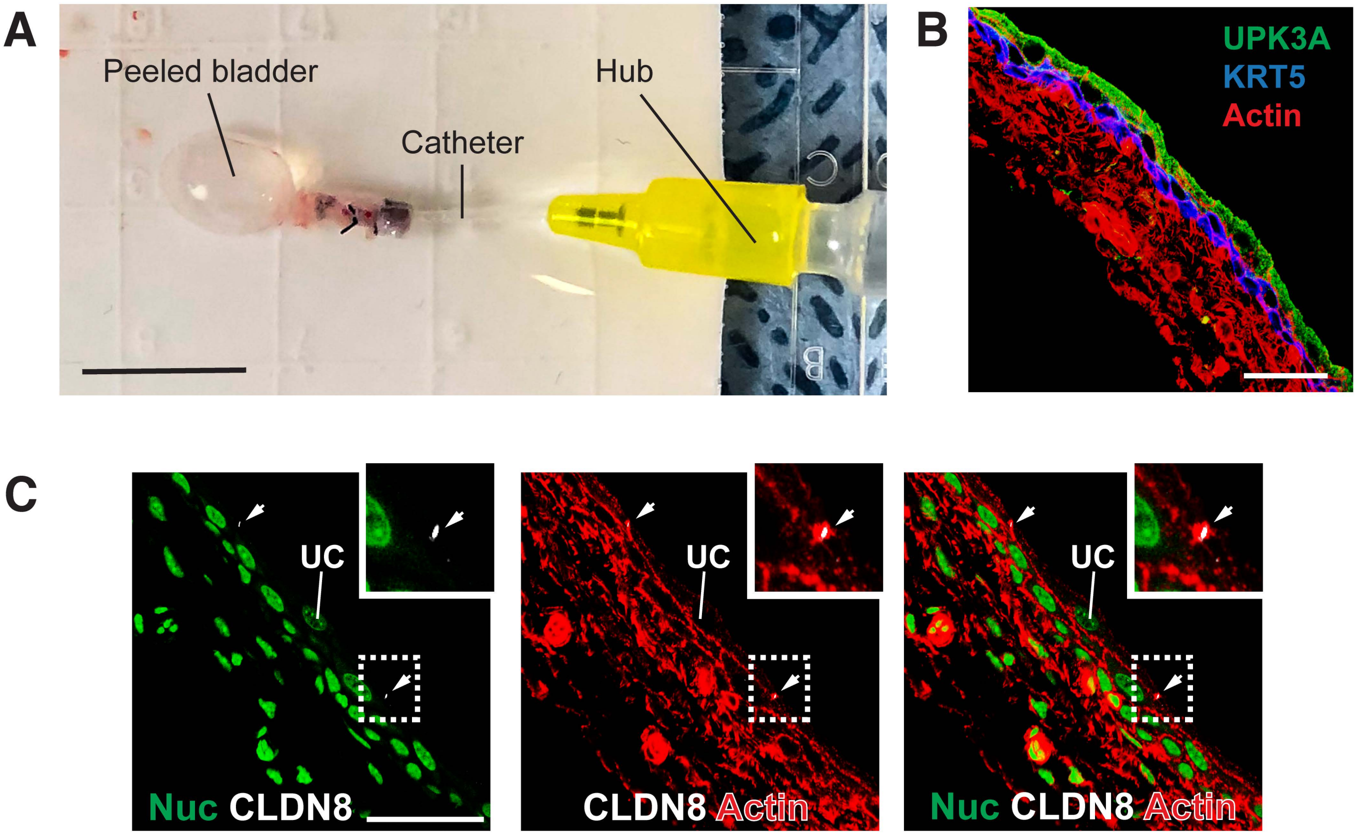

Figure S4. Peeled bladder preparation. **(A)** A peeled bladder is shown attached to a catheter and hub assembly. Scale bar is 1 cm. **(B)** Cross section through peeled bladder preparation, stained with antibodies to UPK3A and KRT5, and counterstained with rhodamine-phalloidin (actin). Image acquired using a confocal microscope and 3D reconstruction from 4 optical slices. Scale bar is 50 μm. **(C)** Distribution of To-Pro-3-labeled nuclei (green), CLDN8 (white), and rhodamine-phalloidin-labeled actin (red). Boxed area surrounded by white dashed line is magnified in the insets. The position of the tight junctions are marked with white arrows. Images, obtained using a confocal microscope, are 3D reconstructions of 6 optical sections. Images in panels B and C were are reproduced from reference (7), with permission from the American Physiological Society. Scale bar is 50 μm.

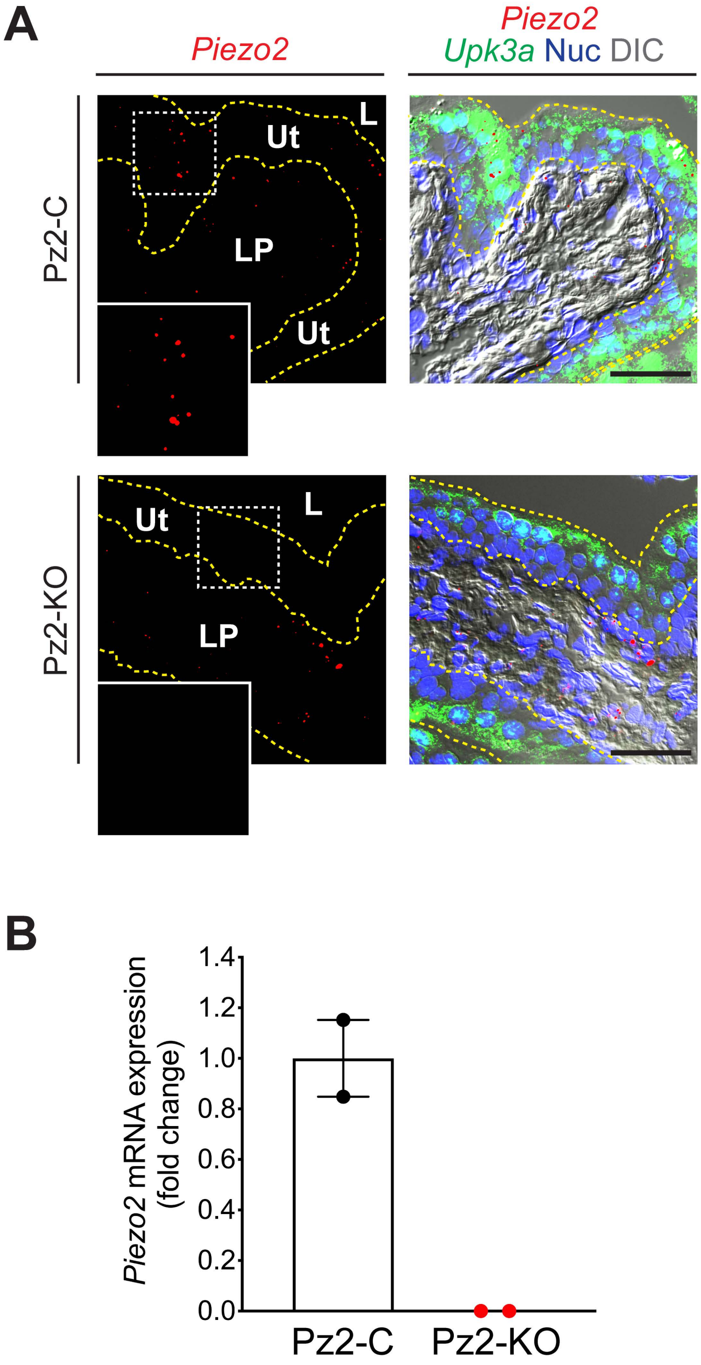

Figure S5. Expression of *Piezo2* in *Piezo2*-control and *Piezo2*-KO mice. **(A)** FISH analysis of *Piezo2* and *Upk3a* expression in the indicated mouse strains. The boxed area surrounded by a white dashed line is magnified in the inset. The boundaries of the urothelium are indicated by yellow dashed lines. Key: DIC, differential interference contrast; L, lumen; LP, lamina propria; Nuc, nucleus; Ut, urothelium. Scale bars are 50 μm. **(B)** The number of *Piezo2*-positive umbrella cells per length of lumen were quantified using FISH and normalized to data obtained for *Piezo2*-control animals. Average values for the indicated mouse strain (n=2).

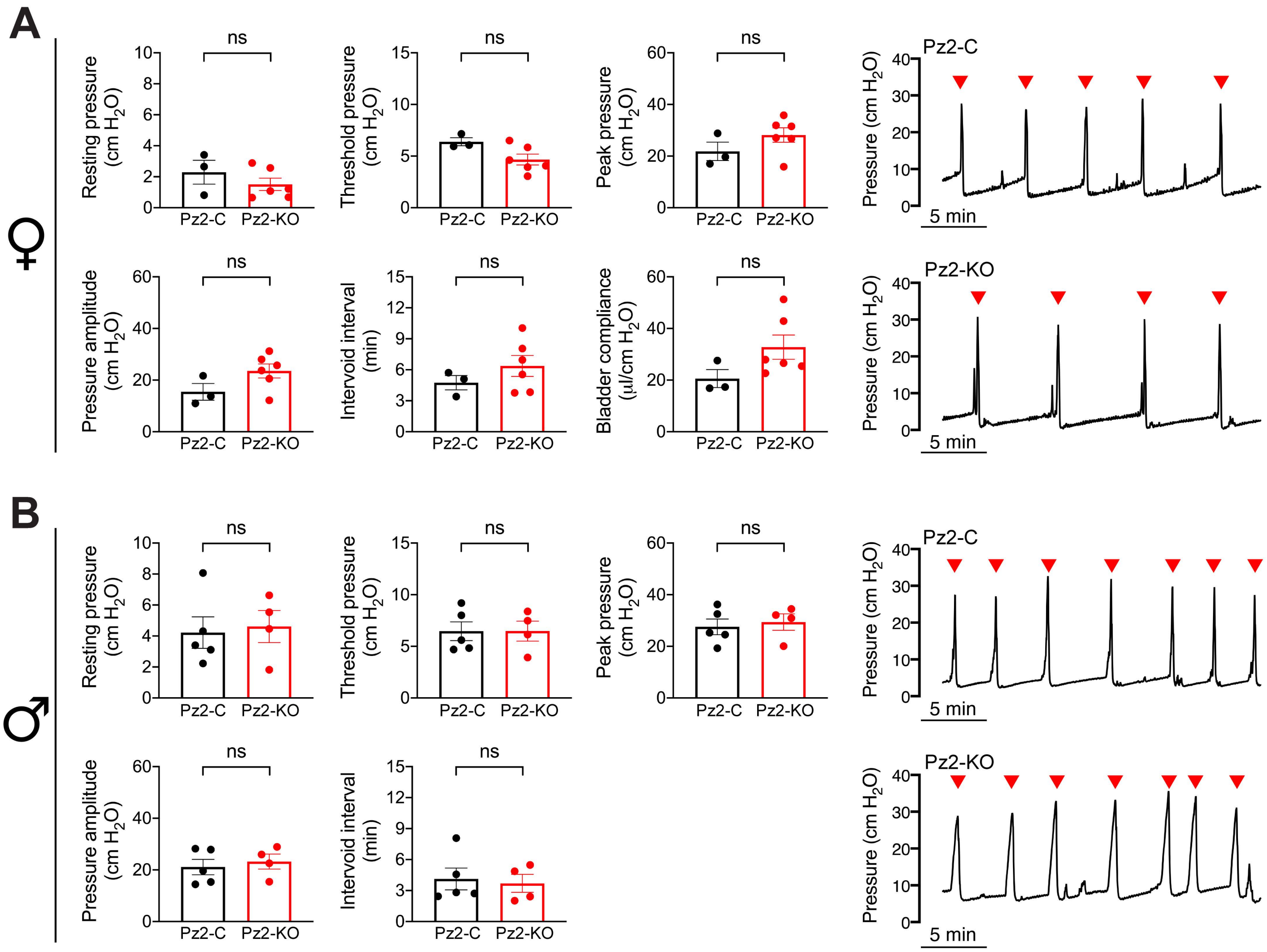

Figure S6. Voiding function of *Piezo2*-control and *Piezo2*-KO mice assessed by cystometry. **(A-B)** Urethane-anesthetized female **(A)** and male **(B)** mice were subjected to cystometry. Representative cystometrograms are shown to the right of the figure. Data are mean ± SEM (female *Piezo2*-control, n=3; female *Piezo2*-KO, n=6; male *Piezo2*-control, n=5; male *Piezo2*-KO, n=4). Data were analyzed using Mann-Whitney tests, but no significant differences were observed.

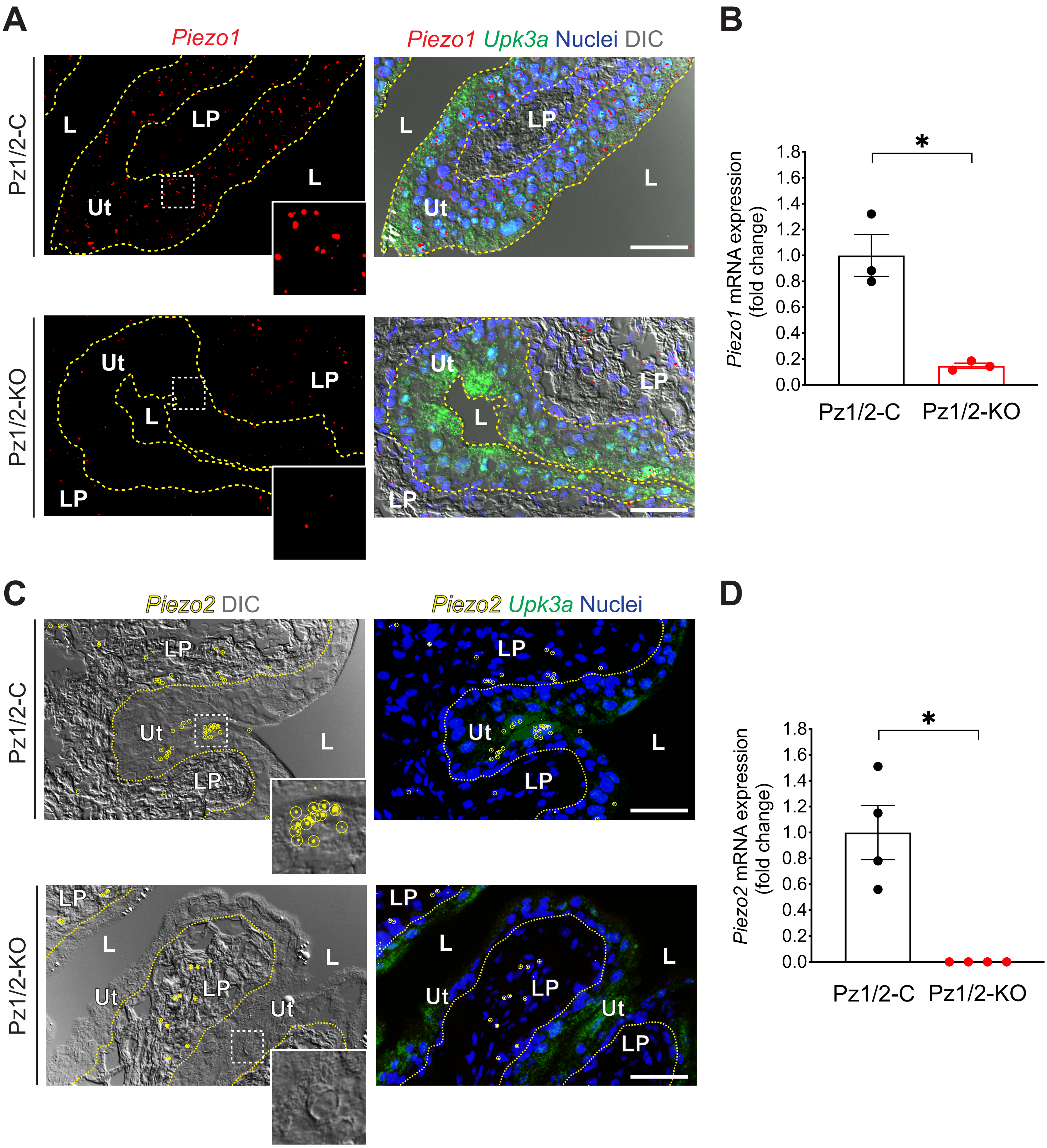

Figure S7. Expression of *Piezo1* and *Piezo2* in *Piezo1/2*-control and *Piezo1/2*-KO mice. **(A)** Expression of *Piezo1* and *Upk3a* in *Piezo1/2*-control or *Piezo1/2*-KO mice was assessed using FISH. The boxed area surrounded by a white dashed line is magnified in the inset. The boundaries of the urothelium are indicated by yellow dashed lines. Key: DIC, differential interference contrast microscopy; L, lumen; LP, lamina propria; Ut, urothelium. **(B)** Expression of *Piezo1* normalized to control values. Data are mean ± SEM (n=3). Data were analyzed using t-tests, and significant differences indicated by an asterisk ($p \leq 0.05$). **(C)** Expression of *Piezo2* and *Upk3a* in *Piezo1/2*-control or *Piezo1/2*-KO mice was assessed using FISH. Signal dots are surrounded by yellow circles. **(D)** Expression of *Piezo2* normalized to control values. Data are mean ± SEM (n=4). Data were analyzed using t-tests, and significant differences indicated by an asterisk ($p \leq 0.05$). All scale bars are 50 μm.

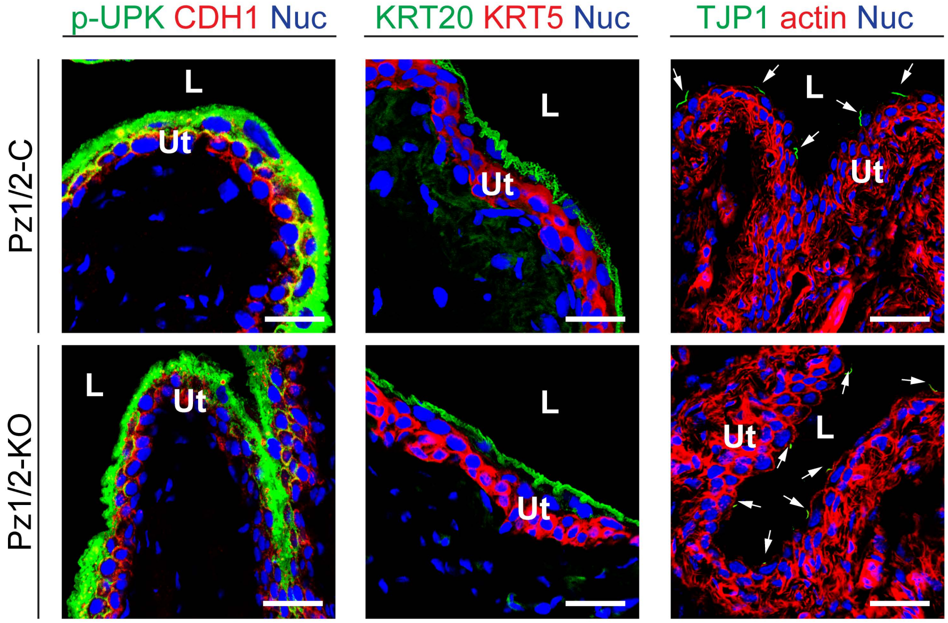

Figure S8. Expression and distribution of urothelial differentiation markers in *Piezo1/2*-control and *Piezo1/2*-KO mouse urothelium. Cryosections were immunostained with a pan-specific antibody that detects multiple uroplakins (p-UPK), or antibodies against CDH1, KRT5, KRT20, or TJP1. Samples were counterstained with rhodamine phalloidin and/or To-Pro-3. Images, obtained using a confocal microscope, are 3D reconstructions of 20-59 optical sections. Key: L = lumen; Ut, urothelium. Scale bars are 40 μm.

Full unedited gel for Figure 1

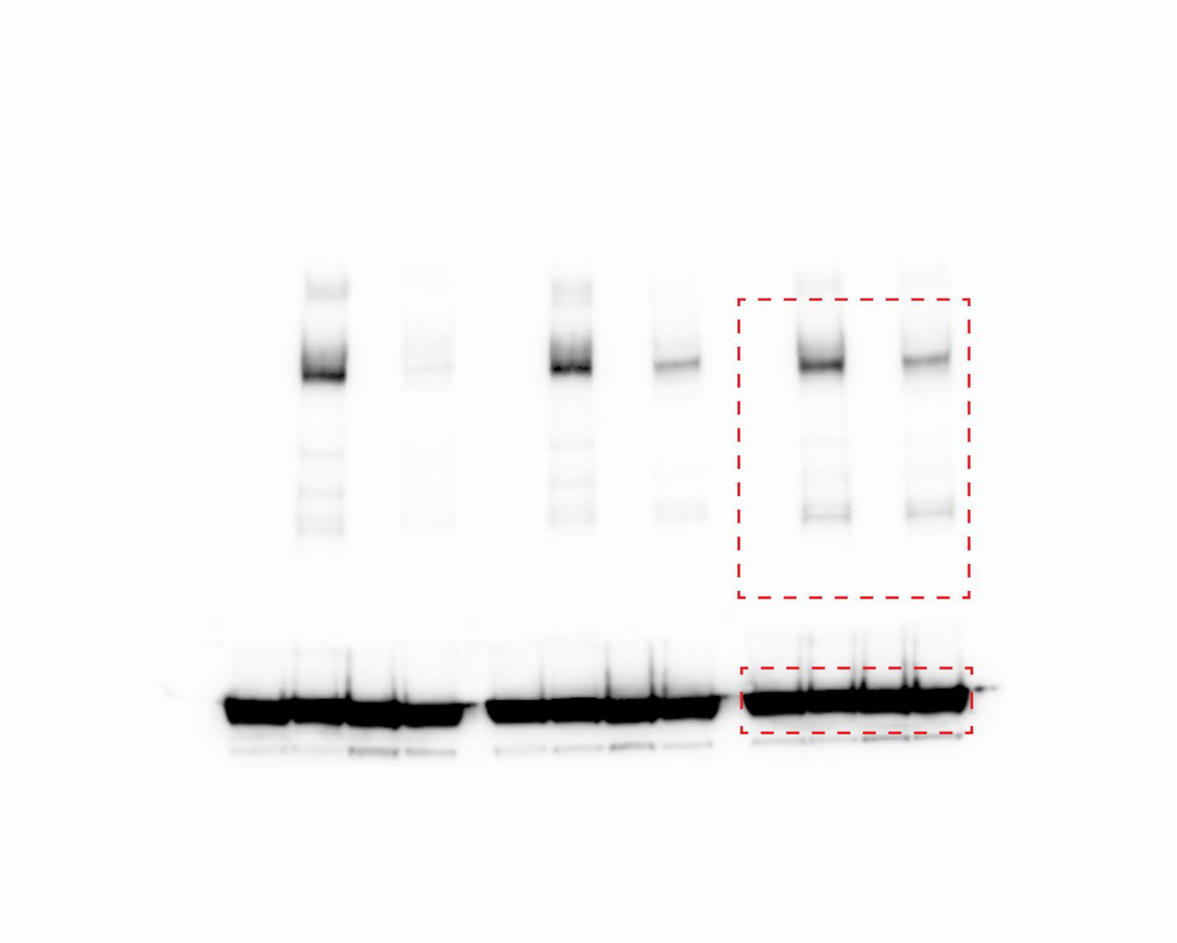

Supplement: Supplemental data [file jciinsight-6-152984-s200.pdf]
